# Trimetallic Sulfide Hollow Superstructures with Engineered d‐Band Center for Oxygen Reduction to Hydrogen Peroxide in Alkaline Solution

**DOI:** 10.1002/advs.202104768

**Published:** 2022-03-01

**Authors:** Chaoqi Zhang, Ruihu Lu, Chao Liu, Jingyi Lu, Yingying Zou, Ling Yuan, Jing Wang, Guozhong Wang, Yan Zhao, Chengzhong Yu

**Affiliations:** ^1^ School of Chemistry and Molecular Engineering East China Normal University Shanghai 200241 P. R. China; ^2^ State Key Laboratory of Silicate Materials for Architectures Wuhan University of Technology Wuhan 430070 China; ^3^ Key Laboratory of Materials Physics Centre for Environmental and Energy Nanomaterials Anhui Key Laboratory of Nanomaterials and Nanotechnology Institute of Solid State Physics Chinese Academy of Sciences Hefei 230031 P. R. China; ^4^ Australian Institute for Bioengineering and Nanotechnology The University of Queensland Brisbane Queensland 4072 Australia

**Keywords:** d‐band center, H_2_O_2_ production, hollow superstructure, oxygen reduction, trimetallic sulfide

## Abstract

High‐performance transition metal chalcogenides (TMCs) as electrocatalysts for two‐electron oxygen reduction reaction (2e‐ORR) in alkaline medium are promising for hydrogen peroxide (H_2_O_2_) production, but their synthesis remains challenging. In this work, a titanium‐doped zinc–cobalt sulfide hollow superstructure (Ti–ZnCoS HSS) is rationally designed as an efficient electrocatalyst for H_2_O_2_ electrosynthesis. Synthesized by using hybrid metal–organic frameworks (MOFs) as precursors after sulfidation treatment, the resultant Ti–ZnCoS HSS exhibits a hollow‐on‐hollow superstructure with small nanocages assembled around a large cake‐like cavity. Both experimental and simulation results demonstrate that the polymetallic composition tailors the d‐band center and binding energy with oxygen species. Moreover, the hollow superstructure provides abundant active sites and promotes mass and electron transfer. The synergistic d‐band center and superstructure engineering at both atomic and nanoscale levels lead to the remarkable 2e‐ORR performance of Ti–ZnCoS HSS with a high selectivity of 98%, activity (potential at 1 mA cm^−2^ of 0.774 V vs reversible hydrogen electrode (RHE)), a H_2_O_2_ production rate of 675 mmol h^–1^ g_cat_^–1^, and long‐term stability in alkaline condition, among the best 2e‐ORR electrocatalysts reported to date. This strategy paves the way toward the rational design of polymetallic TMCs as advanced 2e‐ORR catalysts.

## Graphical abstract

Titanium‐doped zinc–cobalt sulfide hollow superstructure is rationally designed as an efficient two‐electron reduction reaction (2e‐ORR) electrocatalyst. Thanks to the synergistic d‐band center and superstructure engineering at atomic and nanoscale levels, remarkable 2e‐ORR performances with high selectivity, hydrogen peroxide (H_2_O_2_) yield, and stability are achieved.

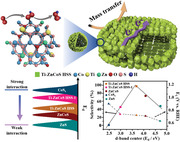

## 1 Introduction

The electrocatalytic two‐electron oxygen reduction reaction (2e‐ORR) presents an emerging route for hydrogen peroxide (H_2_O_2_) production, which only needs O_2_ and H_2_O as sources and electricity as energy input.^[^ ^1^, ^2^, ^3^ ^]^ Compared to the classical anthraquinone process, the 2e‐ORR exhibits many advantages such as improved safety, easy operation, and environmental friendliness.^[^ ^4^, ^5^ ^]^ The key issue during the 2e‐ORR process is the design of efficient electrocatalysts to reduce O_2_ specifically to H_2_O_2_, instead of H_2_O via a competitive 4e‐ORR pathway.^[^ ^6^, ^7^ ^]^ The state‐of‐the‐art 2e‐ORR electrocatalysts are mainly carbon materials,^[^ ^2^, ^7^, ^8^, ^9^, ^10^, ^11^ ^]^ noble metal, and their alloys.^[^ ^12^, ^13^, ^14^ ^]^ For carbon materials, the activity is originated from carbon defects, heteroelement doping, and surface functionalization; however, precise control over defects and dopants at atomic level remains challenging.^[^ ^7^, ^9^, ^10^ ^]^ For noble‐metal‐based catalysts, the formation of alloy could transform the traditional 4e‐ORR catalysts (e.g., Pt and Pd) into the 2e‐process‐favored materials (e.g., Pt—Hg, Pd—Hg, and Au—Hg), which show high selectivity and activity for H_2_O_2_ production.^[^ ^5^, ^12^, ^14^, ^15^ ^]^ However, the cost‐effectiveness and usage of toxic metal (e.g., Hg) hinder their applications.^[^ ^12^, ^14^ ^]^ Therefore, the search of alternative 2e‐ORR electrocatalysts with high performance and reduced cost is still in high demand.

Over the past decades, transition metal chalcogenides (TMCs) have received intensive attention in various fields owing to their earth abundance, versatile redox properties, and structural/compositional diversity.^[^ ^16^ ^]^ A plenty of TMCs have been identified as active catalysts for electrocatalytic reactions such as hydrogen evolution reaction (HER),^[^ ^17^ ^]^ oxygen evolution reaction (OER), and 4e‐ORR;^[^ ^18^ ^]^ however, their application in 2e‐ORR is still in the infancy. A few TMCs including CoS_2_, CoSe_2_, MoTe_2_, and NiS_2_ have been used as 2e‐ORR catalysts with good selectivity and activity in acidic medium,^[^ ^19^, ^20^, ^21^, ^22^ ^]^ but rarely in alkaline conditions. Compared with acidic medium, alkaline electrolyte environment is more favorable for 2e‐ORR due to significantly lower overpotential for the first electron transfer step and higher energy conversion efficiency.^[^ ^23^, ^24^ ^]^ The alkaline medium is also required in water treatment, papermaking, textile industry, and many other important fields related to H_2_O_2_.^[^ ^25^ ^]^ In addition, the alkaline condition is less corrosive and enables the long‐term activity of TMCs.^[^ ^26^ ^]^ Therefore, rational design of TMCs as effective 2e‐ORR electrocatalysts in alkaline condition is highly attractive, yet fairly challenging.

Construction of polymetallic sulfides is a promising strategy to enhance the performance of electrocatalysts.^[^ ^27^, ^28^ ^]^ The synergies among different metal elements can effectively regulate the electronic structure toward optimized adsorption free energies of oxygen intermediates, eventually promoting the activity of electrocatalysis reactions such as OER, HER, and 4e‐ORR.^[^ ^29^, ^30^, ^31^ ^]^ Nevertheless, polymetallic sulfides are seldom applied in 2e‐ORR. In addition to the composition, design of advanced architectures such as hollow superstructures (HSS) assembled by hollow nanoparticles as subunits is also important for boosting the electrocatalytic properties by promoting the active site exposure, mass diffusion, and electron transfer.^[^ ^32^, ^33^ ^]^ However, reported TMCs based 2e‐ORR electrocatalysts are mostly solid and composed of single metal component with much room to improve in their structure and performance.^[^ ^19^, ^20^, ^21^, ^22^ ^]^ Therefore, construction of polymetallic TMC HSSs is expected to be a promising route to prepare highly active, selective, and stable 2e‐ORR electrocatalysts for H_2_O_2_ production.

**Figure 1:**
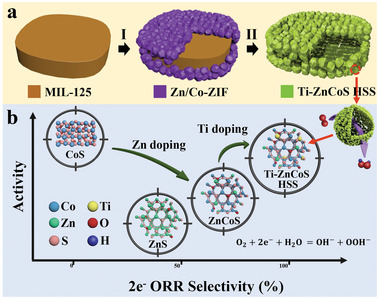
a) A scheme of the two‐step synthesis of Ti–ZnCoS HSS. b) Illustration of the 2e‐ORR activity–selectivity of CoS*_x_*, ZnS, ZnCoS, and Ti–ZnCoS HSS.

Herein, we report the preparation of titanium‐doped zinc cobalt sulfide HSS (Ti–ZnCoS HSS) as an efficient 2e‐ORR electrocatalyst in alkaline media. The two‐step synthetic process of Ti–ZnCoS HSS is schematically illustrated in **Figure** **1**a. Core–shell NH_2_‐MIL‐125@ZnCo‐ZIF (MIL: Matérial Institut Lavoisier, ZIF = zeolite imidazole framework) hybrid metal–organic frameworks (MOFs) were prepared as precursors in the first step by directly growing ZnCo‐ZIF nanocrystals on the surface of NH_2_‐MIL‐125 nanocakes. Through further sulfidation treatment in the second step, ZnCo‐ZIF nanocrystals were converted into interconnected ZnCoS nanocages, while NH_2_‐MIL‐125 nanocakes were etched to form a large cavity. Meanwhile, the decomposed Ti species were partially incorporated into ZnCoS, resulting in trimetallic Ti–ZnCoS HSS with a hollow‐on‐hollow superstructure. Through valence band spectra test and density functional theory (DFT) simulation, it is shown that the polymetallic composite design can adjust the d‐band center of electrocatalysts toward an optimized adsorption energy of oxygen intermediates compared to mono‐ or bimetallic compositions. Moreover, the hollow superstructure led to more active sites and accelerated mass and electron transfer. Therefore, due to the synergistic d‐band center and superstructure engineering, Ti–ZnCoS HSS exhibited remarkable 2e‐ORR performance with a high selectivity of ≈98%, excellent activity (potential at 1 mA cm^−2^ of 0.774 V vs reversible hydrogen electrode (RHE)), and H_2_O_2_ production rate (675 mmol h^–1^ g_cat_^–1^), among the best 2e‐ORR electrocatalysts reported to date (Table S2, Supporting Information). To our best knowledge, the synergistic enhancement at both atomic and nanoscale levels in 2e‐ORR TMC electrocatalysts is rarely reported, which will provide valuable insight into the design of more efficient 2e‐ORR catalysts.

## 2 Results and Discussion

Scanning electron microscopy (SEM) and transmission electron microscopy (TEM) images of NH_2_‐MIL‐125 (Figure S1a,b, Supporting Information) show the uniform disk‐like morphology with smooth surfaces. The length and thickness were measured to be ≈600–800 and ≈200 nm, respectively. The X‐ray diffraction (XRD) pattern (Figure S1c, Supporting Information) shows the characteristic peaks of crystalline NH_2_‐MIL‐125 (CAS# 1309760‐94‐8). By reacting NH_2_‐MIL‐125 with 2‐methylimidazole (2‐MeIM) and Co(NO_3_)_2_⋅6H_2_O/Zn(NO_3_)_2_⋅6H_2_O (mass ratio of 5.7:1; see details in the Supporting Information), bimetallic ZnCo‐ZIF nanocrystals were grown onto the outer surface of NH_2_‐MIL‐125, forming NH_2_‐MIL‐125@ZnCo‐ZIF heterostructures (Figure 1a‐I). As shown in Figure S2a–c (Supporting Information), NH_2_‐MIL‐125@ZnCo‐ZIF demonstrated a core–shell structure, formed by coating ZnCo‐ZIF nanoparticles with a mean diameter of ≈40 nm on the surface of NH_2_‐MIL‐125. The shell thickness and core size were determined to be ≈40 and 700 nm, in agreement with the particle sizes of ZnCo‐ZIF and NH_2_‐MIL‐125. The high angle annular dark‐field scanning TEM (HADDF STEM) and energy‐dispersive X‐ray spectroscopy (EDX) elemental mapping images are shown in Figure S2d–h (Supporting Information), indicating that the Co and Zn elements are mainly distributed on the outer layer while Ti in the inner core region. Compared to pure NH_2_‐MIL‐125, a new group of diffraction peaks corresponding to ZIF (CAS# 59061‐53‐9) are generated in the XRD pattern of NH_2_‐MIL‐125@ZnCo‐ZIF (Figure S3, Supporting Information), indicating the growth of crystalline ZnCo‐ZIF on NH_2_‐MIL‐125.

**Figure 2:**
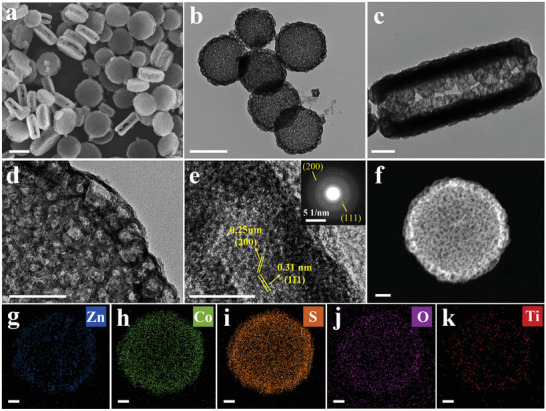
a) SEM, b–d) TEM, e) HRTEM images, and SEAD pattern (inset). f–k) HADDF‐STEM and element mapping images of Ti–ZnCoS HSS. Scale bars are a,b) 500 nm, c,d,f–k) 100 nm, and e) 10 nm.

The resultant NH_2_‐MIL‐125@ZnCo‐ZIF was converted to Ti–ZnCoS HSS through a solvothermal sulfidation treatment at 180 °C for 3 h (Figure 1a‐II). As shown in **Figure** **2**a, the disk‐like morphology of Ti–ZnCoS HSS is well‐retained after the sulfidation treatment. However, TEM images (Figure 2b,c) show the generation of a large hollow cavity with a diameter of ≈700 nm, indicating the removal of NH_2_‐MIL‐125 core. Furthermore, the solid ZnCo‐ZIF nanoparticles were also transformed into hollow nanocages with a cavity size and a shell thickness of ≈20–50 and 6 nm (Figure 2d), respectively, which packed closely around the large disk‐like cavity to form a hollow‐on‐hollow superstructure.

From the high‐resolution TEM (HRTEM) image and selected area electron diffraction (SAED) patterns (Figure 2e and inset), clear lattice fringes and diffraction spots assigned to the (111) and (200) planes of ZnCoS are observed, revealing a polycrystalline nature. The diffraction peaks at 2*θ* values of 28.5°, 47.5°, and 56.3° in the XRD pattern (Figure S4, Supporting Information) well match the simulated ZnCoS (JCPDS No. 47–1655).^[^ ^34^ ^]^ The HAADF STEM and EDX element mapping images show the homogeneous distribution of Zn, Co, S, O, and Ti elements in the framework of Ti–ZnCoS HSS (Figure 2f–k). Inductive coupled plasma emission spectrometer (ICP) was used to quantify the Zn/Co/Ti ratio, which was determined to be 1/3.8/1 (Table S1, Supporting Information).

The specific surface area and pore structure of Ti–ZnCoS HSS were determined by N_2_ sorption measurement. The N_2_ sorption isotherms (Figure S5a, Supporting Information) demonstrate the characteristics of type IV isotherms with a major capillary condensation step occurred at *P*/*P*_0_ = 0.85–0.99, revealing the existence of large mesopores. The pore size distribution curve (Figure S5b, Supporting Information) derived from the Barrett–Joyner–Halenda (BJH) model shows mesopores with a mean diameter centered at ≈20 nm, close to the cavity size of hollow nanocage, suggesting that the mesopores are mainly originated from the hollow subunits derived from the solvothermal sulfidation of ZnCo‐ZIF nanoparticles. The Brunauer–Emmett–Teller (BET) specific surface area and total pore volume are calculated to be 142.3 m^2^ g^–1^ and 0.51 cm^3^ g^–1^, respectively.

Afterward, the structure evolution of NH_2_‐MIL‐125@ZnCo‐ZIF as a function of the sulfidation time was investigated. With a reaction for 0.5 h, the solid ZnCo‐ZIF nanocrystals were converted into interconnected hollow nanocages, and the interior NH_2_‐MIL‐125 was partially etched with the diameter decreasing from 700 to 550 nm (Figure S6a, Supporting Information). The XRD (Figure S6d, Supporting Information) pattern demonstrates the disappearance of diffraction peaks of Zn, Co‐ZIF due to its conversion into metal sulfide. Meanwhile, the peak intensity of NH_2_‐MIL‐125 is significantly weakened. After 1 h, the size of NH_2_‐MIL‐125 further decreased to 440 nm (Figure S6b, Supporting Information). By further prolonging the time to 5 h, the superstructure was destroyed (Figure S6c, Supporting Information). In addition, the effect of sulfidation temperature was also explored at a reaction time of 3 h. As shown in Figure S7a,b,d (Supporting Information), the NH_2_‐MIL‐125 core cannot be completely removed when decreasing the temperature to 100 or 140 °C. By increasing the temperature to 220 °C, the superstructure was seriously damaged. Therefore, the optimized sulfidation condition is determined to be 180 °C for 3 h for the formation of Ti–ZnCoS HSS.

**Figure 3:**
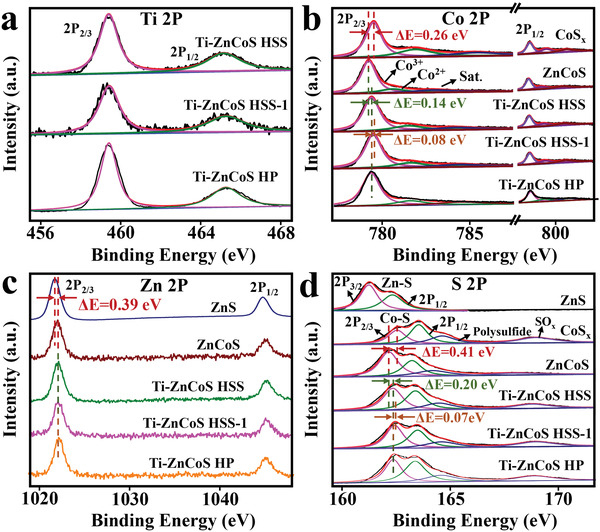
High‐resolution XPS spectra of a) Ti 2p, b) Co 2p, c) Zn 2p, and d) S 2p of ZnS, CoS*_x_*, ZnCoS, Ti–ZnCoS HSS, Ti–ZnCoS HSS‐1, and Ti–ZnCoS HP.

For comparison, zinc sulfide (ZnS), cobalt sulfide (CoS*_x_*), and zinc–cobalt sulfide (ZnCoS) were prepared by the same solvothermal sulfidation treatment of corresponding ZIF precursors (ZIF‐8, ZIF‐67, and ZnCo‐ZIF). Crystalline ZnS, ZnCoS, and amorphous CoS*_x_* were obtained as evidenced by TEM, SEM, and XRD analyses (Figures S8–S13, Supporting Information). Furthermore, by changing the feeding mass ratio of Co(NO_3_)_2_⋅6H_2_O/Zn(NO_3_)_2_⋅6H_2_O into 19/1 while keeping the other conditions the same as Ti–ZnCoS HSS, Ti–ZnCoS HSS‐1 with a similar superstructure was also fabricated (Figures S14 and S15, Supporting Information). In order to demonstrate the advantages of hollow superstructure, isolated titanium‐doped zinc–cobalt sulfide hollow particles (Ti–ZnCoS HP) without superstructure were also synthesized via treating the mixture of ZnCo‐ZIF and NH_2_‐MIL‐125. The Co/Zn/Ti ratio of Ti–ZnCoS HP was measured to be consistent with Ti–ZnCoS HSS (Figure S16 and Table S1, Supporting Information). The BET specific surface area and pore volume of Ti–ZnCoS HP were calculated to be 65.8 m^2^ g^–1^ and 0.21 cm^2^ g^–1^ (Figure S17, Supporting Information), respectively, lower than those of Ti–ZnCoS HSS. The surface chemical structures of all synthesized metal sulfides were investigated by X‐ray photoelectron spectroscopy (XPS). As presented in Figure S18 (Supporting Information), Co, Zn, S, O, and Ti elements were detected in the survey scan XPS spectra of Ti–ZnCoS HSS, Ti–ZnCoS HP, and Ti–ZnCoS HSS‐1 with a similar Ti content (1.3% for Ti–ZnCoS HSS, 1.4% for Ti–ZnCoS HP, and 1.5% for Ti–ZnCoS HSS‐1). The Co/Zn ratios of Ti–ZnCoS HSS (5.7/1) and Ti–ZnCoS HP (5.6/1) are similar, while increase to 7.5/1 for Ti–ZnCoS HSS‐1. The typical peaks at 459.41 and 465.16 eV in the Ti 2p spectra of these three samples are assigned to Ti 2p_3/2_ and 2p_1/2_ of Ti (IV) (**Figure** **3**a).^[^ ^35^ ^]^ In contrast, no Ti signal can be observed in ZnS, CoS*_x_*, and ZnCoS hollow particles.

The electronic structures of Co, Zn, and S elements in different metal sulfides were also compared. The Co 2p spectrum of CoS*_x_* is shown in Figure 3b, which can be fitted into Co 2p_1/2_ and Co 2P_3/2_ orbitals of Co^3+^ (794.35 and 779.43 eV), Co^2+^ (797.15 and 781.53 eV), and satellite peaks (802.15 and 784.33 eV) with Co^3+^/Co^2+^ ratio of 2.2/1. Compared with CoS*_x_*, the binding energy of Co 2P_3/2_ orbital of Co^3+^ in ZnCoS (779.17 eV) presents a negative shift of 0.26 eV and the Co^3+^/Co^2+^ ratio increases to 4.7/1, similar to the reported Zn‐doped cobalt compounds.^[^ ^36^, ^37^ ^]^ In contrast, by further incorporating Ti element, the Co 2P_3/2_ peak (779.31 eV) shifts toward higher binding energy, and the Co^3+^/Co^2+^ ratio decreases from 4.7/1 to 3.7/1 due to the electron density shift from Ti to Co center,^[^ ^38^, ^39^ ^]^ which locates between ZnCoS and CoS*_x_*. When increasing the Co/Zn ratio, the binding energy of Co 2P_3/2_ (≈779.39 eV) in Ti–ZnCoS HSS‐1 further increases. With almost the same Co/Zn ratio and Ti content, the binding energies of Co in Ti–ZnCoS HP were also similar to that of Ti–ZnCoS HSS.

The Zn 2p spectrum of ZnS (Figure 3c) shows two peaks at 1021.73 and 1044.79 eV, attributed to Zn 2P_3/2_ and 2p_1/2_, respectively. Compared to ZnS, the binding energy of Zn 2P_3/2_ peak (1022.12 eV) in ZnCoS positively shifts by ≈0.39 eV, further indicating the electronic interaction between Zn and Co.^[^ ^40^ ^]^ However, negligible change of Zn 2p peaks in Ti–ZnCoS HSS, Ti–ZnCoS HSS‐1, and Ti–ZnCoS HP was observed compared with ZnCoS. The Ti doping and change in ratio of Co^2+^/Zn^2+^ have little effect on the electronic structure of Zn, which may be attributed to the fully occupied 3d^10^ electronic configuration of Zn^2+^.^[^ ^40^, ^41^ ^]^

The S 2p spectrum of ZnS can be divided into two peaks at 161.24 and 162.35 eV, corresponding to the S 2p_3/2_ and S 2p_1/2_ of Zn—S bond. For CoS*_x_*, there are four peaks at 162.54, 163.53, and 164.65, ascribed to 2p_3/2_ and 2p_1/2_ of Co—S bond and polysulfide, respectively (Figure 3d).^[^ ^42^ ^]^ The peak at 168.99 eV can be attributed to the oxidized forms of sulfur (SO*_x_*),^[^ ^43^ ^]^ which arises from the partial oxidation of sulfur‐containing compounds during the hydrothermal process. Compared with CoS*_x_*, the binding energy of S 2p_3/2_ in ZnCoS negatively shifts by ≈0.41 eV. However, this peak in the spectrum of Ti–ZnCoS HSS shows a positive shift of ≈0.20 eV with further titanium doping. For Ti–ZnCoS HSS‐1, the binding energy of S 2p_3/2_ peak further increases by ≈0.07 eV. The change trend of S 2p is in agreement with Co 2p. Together with the unchanged binding energy of Zn 2p of Ti–ZnCoS HSS compared with ZnCoS, the above results presumably indicate the formation of Ti—S—Co and Zn—S—Co bonds in Ti–ZnCoS HSS. There is no obvious difference of S 2p spectra among Ti–ZnCoS HSS, Ti–ZnCoS HSS‐1, and Ti–ZnCoS HP, similar to the Zn 2p in Figure 3c.

**Figure 4:**
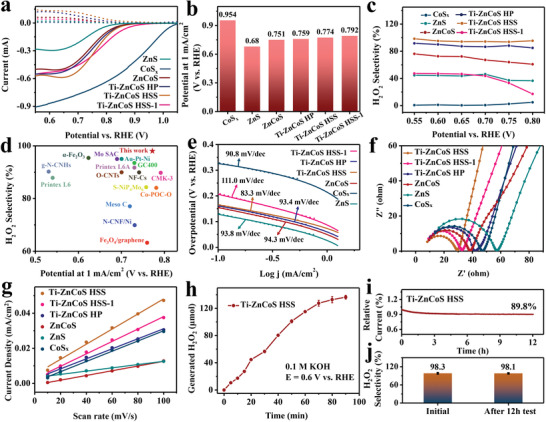
a) LSV polarization curves, dashed and solid lines are current on Pt‐ring (*i*_r_) and disk (*i*_d_), respectively. b) Onset potentials and c) H_2_O_2_ selectivity of CoS*_x_*, ZnS, ZnCoS, Ti–ZnCoS HP, Ti–ZnCoS HSS, and Ti–ZnCoS HSS‐1 in O_2_ saturated 0.1 m KOH solution. d) Comparison of the activity and selectivity (at 0.55 V vs RHE) for H_2_O_2_ electrosynthesis on Ti–ZnCoS HSS and other recently reported electrocatalysts. e) Tafel slope curves, f) EIS spectra, and g) CV current density versus scan rate; the linear slope is equivalent to the double‐layer capacitance (*C*_dl_) of CoS*_x_*, ZnS, ZnCoS, Ti–ZnCoS HP, Ti–ZnCoS HSS, and Ti–ZnCoS HSS‐1. h) H_2_O_2_ concentration versus time. i) Long‐term stability test. j) H_2_O_2_ selectivity after 12 h measurement of Ti–ZnCoS HSS at 0.55 V versus RHE.

The electrochemical 2e‐ORR performance of Ti–ZnCoS HSS was evaluated using a three‐electrode system in 0.1 m KOH solution (see the details in the “Experimental Section” of the Supporting Information) in comparison with ZnS, CoS*_x_*, ZnCoS, Ti–ZnCoS HSS‐1, and Ti–ZnCoS HP. The liner sweep voltammetry (LSV) curves of different electrocatalysts were recorded using a rotating ring‐disk electrode (RRDE) at 1600 rpm, where ORR took place at the disk electrode and the generated H_2_O_2_ was oxidized at the ring electrode subsequently. The collection efficiency (*N*) was determined to be 0.258 by using the standard ferricyanide system (Figure S19, Supporting Information). As can be seen in **Figure** **4**a, CoS*_x_* exhibited the highest onset potential at 1 mA cm^−2^ (*E*_1_) of 0.954 V (Figure 4b), indicative of the highest activity, but the lowest selectivity of ≈1% for 2e‐ORR (Figure 4c). For ZnS, the activity was obviously lower with *E*_1_ of 0.680 V, while the selectivity was higher (≈48%). The activity of ZnCoS (*E*_1_ = 0.751 V) was higher than ZnS, but ≈200 mV lower than CoS*_x_*. However, its selectivity increased to ≈73%, suggesting the positive contribution of zinc doping on selectivity but negative contribution on activity for 2e‐ORR.

Unexpectedly, a significant improvement on selectivity to ≈98% was achieved for Ti–ZnCoS HSS, superior to other prepared metal sulfides and even among the best electrocatalysts reported to date (Figure 4d; Table S2, Supporting Information). Simultaneously, however, increasing the Co/Zn ratio from 5.7/1 in Ti–ZnCoS HSS to 7.5/1 in Ti–ZnCoS HSS‐1 dramatically decreased the selectivity to ≈49%. The electron transferred number (*n*) of Ti–ZnCoS HSS was calculated to be 2.04 at 0.55 V versus RHE, indicating that the ORR over Ti–ZnCoS HSS was closer to a 2e^–^ pathway compared with CoS*_x_* (*n* = 3.87), ZnS (*n* = 3.09), ZnCoS (*n* = 2.70), and Ti–ZnCoS HSS‐1 (*n* = 3.06). The above results reveal that the titanium doping and an appropriate ratio of Zn^2+^/Co^2+^ are beneficial for 2e‐ORR toward both higher activity and selectivity. In addition, the performance of Ti–ZnCoS HSS also outperforms Ti–ZnCoS HP with the *E*_1_ of 0.759 V and selectivity of ≈90%, revealing the positive contribution of superstructure.

Furthermore, the solid H_2_O_2_ powder (inset in Figure S20 of the Supporting Information) could be extracted from the reaction media after electrolysis based on the following reaction: Na_2_CO_3_ + 1.5H_2_O_2_ → Na_2_CO_3_∙1.5H_2_O_2_ (see the details in the Supporting Information). As shown in Figure S20 (Supporting Information), the characteristic peaks of Na_2_CO_3_∙1.5H_2_O_2_ (PDF No. 11–0656) were observed in the XRD pattern, indicating the successful extraction of H_2_O_2_ from solution into a solid form with improved stability.^[^ ^44^ ^]^ As a simple demonstration, the as‐prepared H_2_O_2_ powder can be directly used as an efficient oxidant for water treatment. Degradation of methylene red (MA), methylene blue (MB), and methylene orange (MO) was chosen as the model reactions. As shown in the inset photograph in Figure S20 (Supporting Information), the organic dyes were totally decolorized by adding the solid Na_2_CO_3_∙1.5H_2_O_2_ powder, showing its high oxidation ability.

To gain insight into the enhanced performance of Ti–ZnCoS HSS, the Tafel slopes of different samples were measured to determine the reaction kinetics. Among all samples, Ti–ZnCoS HSS exhibited a smaller Tafel slope of 83.3 mV dec^–1^ than ZnS (93.8 mV dec^–1^), CoS*_x_* (90.8 mV dec^–1^), ZnCoS (94.3 mV dec^–1^), Ti–ZnCoS HSS‐1 (111.0 mV dec^–1^), and Ti–ZnCoS HP (93.4 mV dec^–1^), indicating faster 2e‐ORR kinetics of Ti–ZnCoS HSS (Figure 4e). Electrochemical impedance spectra (EIS) were also recorded to evaluate the charge‐ and mass‐transporting ability of the above samples. As shown in Figure 4f, Ti–ZnCoS HSS showed the smallest diameter of the semicircle, suggesting the lowest charge‐transfer resistance (*R*_ct_). Meanwhile, the largest slash slope of Ti–ZnCoS HSS suggests a fast ion/charge diffusion at the electrode–electrolyte interface (Table S3, Supporting Information). In addition, electrochemical active surface area (ECSA) was evaluated based on the double‐layer capacitances (*C*_dl_) of the catalysts, which were derived from cyclic voltammograms (CV) at different voltage scanning rates (Figure S21, Supporting Information). The results show that the *C*_dl_ of Ti–ZnCoS HSS (0.42 mF cm^−2^) is higher than that of ZnS (0.09 mF cm^−2^), CoS*_x_* (0.29 mF cm^−2^), ZnCoS (0.13 mF cm^−2^), Ti–ZnCoS HSS‐1 (0.35 mF cm^−2^), and Ti–ZnCoS HP (0.28 mF cm^−2^), implying that the active site exposure is improved (Figure 4g).

The cumulative H_2_O_2_ yield measurement of Ti–ZnCoS HSS was conducted in H‐type electrolytic cell with Ag/AgCl (3.5 m KCl) as the reference electrode, graphite rod as the counter electrode, and the Ti–ZnCoS HSS supported on carbon fiber paper as the working electrode (1 cm×1.3 cm). The Nafion 117 membrane has widely been used in basic media for separating electrolytic cells, preventing the flow of generated H_2_O_2_ to anode from oxidation.^[^ ^2^, ^7^, ^26^, ^32^, ^44^ ^]^ Figure 4h shows the fast‐liner increase of H_2_O_2_ generation in the first 1 h, then gradually deviated from linearity due to the reduction and/or decomposition of generated H_2_O_2_. The production rate was calculated to be 675 mmol h^–1^ g_cat_^–1^, which is comparable to the reported best catalysts.^[^ ^9^, ^19^, ^20^ ^]^ The Faraday efficiency was further measured using chronoamperometry during 60 min bulk electrolysis. Aliquots of electrolyte were collected from the cathode compartment every 10 min for detection of H_2_O_2_. Figure S22 (Supporting Information) shows that Ti–ZnCoS HSS exhibited a cumulative Faraday efficiency above 95% during 1 h continuous test, further confirming its high selectivity for 2e‐ORR. The measured Faraday efficiency is slightly lower than the selectivity calculated by RRDE, which may due to the partial decomposition of H_2_O_2_ by the Nafion membrane.^[^ ^26^, ^31^ ^]^ Besides, Ti–ZnCoS HSS possesses superior stability with only ≈10.2% decrease of current density after the 12 h chronoamperometric test at 0.6 V versus RHE (Figure 4i). Moreover, the outstanding selectivity of Ti–ZnCoS HSS could be well maintained at ≈98.1% after 12 h test (Figure 4j). Even after 2500 CV cycles, the polarization curve of Ti–ZnCoS HSS shows only ≈4 mV decline of half‐wave potential compared with the initial cycle (Figure S23, Supporting Information), further indicating the electrochemical robustness in the alkaline solutions.

Ti–ZnCoS HSS after the durability test was further characterized by TEM and XRD (Figure S24a,b, Supporting Information). Negligible changes on morphology and crystal structure were found, revealing its robust electrochemical stability. XPS spectra of used Ti–ZnCoS HSS showed no obvious change of Ti and Zn (Figure S24c, Supporting Information). In the Co 2p spectrum, the binding energies of Co^3+^ and Co^2+^ slightly increased, which may be ascribed to the formation of metal hydroxides on the surface of electrocatalyst. Simultaneously, the content of SO*_x_* bands also increased in the S 2p spectrum (Figure S24d, Supporting Information), which may be attributed to the surface oxidation by O_2_ during the process of ORR.^[^ ^45^ ^]^

In general, the 2e‐ORR process in alkaline solution involves the adsorption of O_2_, and electrochemical reduction into OOH* and finally OOH^–^. The activity and selectivity of 2e‐ORR are strongly related to the binding ability of OOH* by electrocatalysts. For an ideal 2e‐ORR catalyst, the adsorption of OOH* should be neither too strong nor too weak to enable a high selectivity and activity. When the interaction is too weak, O_2_ protonation and formation of OOH* intermediate are restricted; thus, both the activity and selectivity are limited. However, too strong interaction may favor the competitive 4e‐ORR with H_2_O instead of H_2_O_2_ as the final product.^[^ ^5^, ^12^, ^13^, ^46^ ^]^ It is widely reported that the binding ability of OOH* can be predicted by the absorption free energy (Δ*G*_OOH*_),^[^ ^2^, ^5^, ^47^ ^]^ which is positively correlated to the d‐band center (*E*_d_, the local average of the d‐band electron energies) in transition metal‐based catalysts.^[^ ^48^, ^49^, ^50^ ^]^

**Figure 5:**
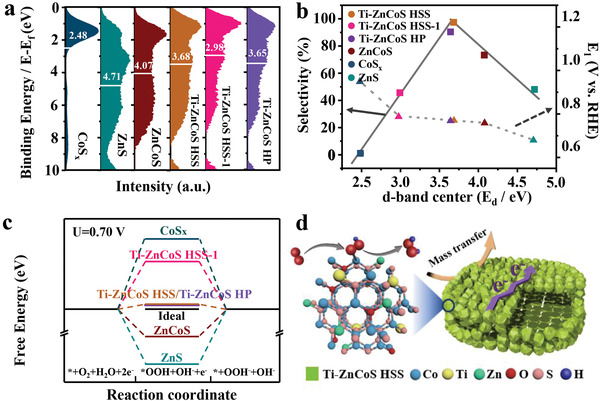
a) Valence band spectra (the white bar shows d‐band center). b) Relationship between selectivity (at 0.55 V vs RHE) and activity (potential at 1 mA cm^–2^) and d‐band center for different catalysts. c) Free‐energy diagram for oxygen reduction to H_2_O_2_. d) Schematic illustration for the 2e‐ORR process of Ti–ZnCoS HSS catalyst.

To understand the structure–performance relationship, the *E*_d_ of different samples was first determined by XPS valence band spectra (VBS, see the details in the “Experimental Section” in the Supporting Information).^[^ ^51^, ^52^, ^53^ ^]^ As shown in **Figure** **5**a, *E*_d_ were calculated to be 2.48, 4.71, 4.07, 3.68, 2.98, and 3.65 eV for CoS*_x_*, ZnS, ZnCoS, Ti–ZnCoS HSS, Ti–ZnCoS HSS‐1, and Ti–ZnCoS HP, respectively. To understand the impact of *E*_d_ on the electrocatalytic performance, both the catalyst activity (represented by *E*_1_) and selectivity were plotted against *E*_d_. As shown in Figure 5b, *E*_l_ increases with *E*_d_ (the dotted line), indicating that stronger interaction (CoS*_x_* > Ti–ZnCoS HSS‐1 > Ti–ZnCoS HSS > ZnCoS > ZnS) favors the ORR activity. Nevertheless, the selectivity and *E*_d_ plot displays a volcano‐type curve (solid line, Figure 5b). Ti–ZnCoS HSS with the highest selectivity close to the peak of the volcano possesses an intermediate *E*_d_ of 3.68 eV. The selectivity of other catalysts decreases with the separation of their *E*_d_ values away from this peak, either with larger or smaller *E*_d_ values.

To further explore the impact of *E*_d_ on adsorption of OOH*, DFT simulation was employed to calculate the Δ*G*_OOH*_. The established energy optimization models of the catalysts are presented in Figure S25 (Supporting Information). The calculated Δ*G*_OOH*_ values on the (200) facets follow the order of CoS*_x_* (3.93 eV) < Ti–ZnCoS HSS‐1 (4.04 eV) < Ti‐ZnCoS HSS (4.19 eV) < ZnCoS (4.83 eV) < ZnS (5.96 eV), showing the same trend with *E*_d_ (Figure S26, Supporting Information). In other words, with the increase of *E*_d_, the Δ*G*_OOH*_ increases, and 2e‐ORR activity decreases. Figure 5c shows the absorption free energy diagram of elementary 2e‐ORR step at the standard potential of 0.7 V on CoS*_x_*, ZnS, ZnCoS, Ti–ZnCoS HSS, and Ti–ZnCoS HSS‐1 catalysts.^[^ ^3^, ^12^, ^22^ ^]^ Compared with CoS*_x_*, Δ*G*_OOH*_ of ZnCoS increased from 3.93 to 4.83 eV, suggesting that the doped zinc weakened the adsorption of OOH* intermediate by decreasing the Co^2+^ content.^[^ ^54^ ^]^ After further doping with titanium, the obtained Ti–ZnCoS HSS exhibits the smallest Δ*G*_OOH*_ deviation of ≈0.03 eV from the ideal value (Δ*G*_OOH*_ = 3.52 eV)^[^ ^3^, ^12^ ^]^ among all samples, in accordance with its highest selectivity.

Collectively, both experimental and DFT simulation results have shown that, for trimetallic TiZnCo TMCs prepared in this study, their absorption energy of intermediate can be regulated by tuning the d‐band center, and thus the performance of the catalysts is modulated (Figure 5d). Ti–ZnCoS HSS shows an *E*_d_ value of 3.68 eV with an appropriate Δ*G*_OOH*_ of 4.19 eV, delivering the highest 2e‐ORR selectivity of 98% together with a reasonably high *E*_1_ of 0.774 V. For Ti–ZnCoS HSS‐1 with similar structure, the relatively lower *E*_d_ (2.98 eV) results in the smaller Δ*G*_OOH_* (4.04 eV) and too strong adsorption of OOH*. This may facilitate the cleavage of O—O bonds to produce H_2_O, thereby leading to the lower 2e^−^ selectivity (49%). Therefore, the choice of a trimetallic TMC system via zinc and titanium element doping is extremely important in our design by finely tuning the *E*_d_ and Δ*G*_OOH*_ close to the ideal state. Meanwhile, the comparison of Ti–ZnCoS HP and Ti–ZnCoS HSS demonstrates the positive role of superstructure. The higher specific surface area and pore volume of Ti–ZnCoS HSS (142.3 m^2^ g^–1^ and 0.51 cm^3^ g^–1^; Figure S5, Supporting Information) than Ti–ZnCoS HP (65.8 m^2^ g^–1^ and 0.21 cm^3^ g^–1^; Figure S17, Supporting Information) may promote the active site exposure, which is further supported by the larger ECSA (Figure 4g). The desorption of H_2_O_2_ may be simultaneously facilitated, avoiding the further oxidation of produced H_2_O_2_ and thus improving the selectivity.^[^ ^9^, ^55^ ^]^ In addition, the smaller electrochemical impedance (Figure 4f) of Ti–ZnCoS HSS than Ti–ZnCoS HP demonstrates the promoted electron transfer by enhancing the interconnection between hollow subunits in superstructure.^[^ ^56^, ^57^, ^58^ ^]^ The introduction of titanium may also enhance the conductivity of the catalyst.^[^ ^59^, ^60^ ^]^ In addition, because the fully occupied Zn^2+^ 3d^10^ electronic configuration can increase the number of electrons occupied in the d orbitals of Co^2+^ in the polymetallic composite,^[^ ^36^, ^37^, ^40^, ^61^ ^]^ presumably the regulation of electronic structure also contributes to the excellent stability of Ti–ZnCoS HSS in alkaline condition. Therefore, the rational design of Ti–ZnCoS HSS with a synergism between d‐band center engineering and superstructure results in the overall best H_2_O_2_ production performance.

## 3 Conclusion

In summary, trimetallic (TiZnCo) TMCs with a hollow superstructure have been synthesized as an electrocatalyst for 2e‐ORR. Experimental findings combined with simulation demonstrate that the polymetallic composition tailors the d‐band center and the absorption energy of intermediate. Furthermore, the hollow superstructure promotes active site exposure, mass diffusion, and electron transfer. The synergistic enhancement leads to the remarkable 2e‐ORR performance of Ti–ZnCoS HSS with an excellent selectivity of ≈98%, an activity of 0.774 V versus RHE at 1 mA cm^−2^, a H_2_O_2_ production rate of 675 mmol h^–1^ g_cat_^–1^, and robust stability in alkaline condition. Our strategies may be applied in the designed synthesis of high‐performance TMCs as electrocatalysts for the 2e‐ORR process.

## Conflict of Interest

The authors declare no conflict of interest.

## Supporting information

Supporting Information

## Data Availability

The data that support the findings of this study are available from the corresponding author upon reasonable request.
