# Supplementary material for: Trimetallic Sulfide Hollow Superstructures with Engineered d‐Band Center for Oxygen Reduction to Hydrogen Peroxide in Alkaline Solution
Source: Adv Sci (Weinh). 2022 Mar 1;9(12):2104768. doi: 10.1002/advs.202104768 (PMC9036009; doi:10.1002/advs.202104768)
Supplement: Supplementary file 1 — Supporting Information [file ADVS-9-2104768-s001.pdf]

ADVANCED SCIENCE
Open Access

Supporting Information

for *Adv. Sci.*, DOI 10.1002/advs.202104768

Trimetallic Sulfide Hollow Superstructures with Engineered d-Band Center for Oxygen Reduction to Hydrogen Peroxide in Alkaline Solution

Chaoqi Zhang, Ruihu Lu, Chao Liu, Jingyi Lu, Yingying Zou, Ling Yuan, Jing Wang, Guozhong Wang, Yan Zhao* and Chengzhong Yu**

WILEY-VCH

ADVANCED SCIENCE
Open Access

Supporting Information

for *Adv. Sci.*, DOI: 10.1002/advs.202104768

Trimetallic Sulfide Hollow Superstructures with Engineered d-Band Center for Oxygen Reduction to Hydrogen Peroxide in Alkaline Solution

Chaoqi Zhang, Ruihu Lu, Chao Liu, Jingyi Lu, Yingying Zou, Ling Yuan, Jing Wang, Guozhong Wang, Yan Zhao, * Chengzhong Yu**

Supporting Information

Trimetallic Sulfide Hollow Superstructures with Engineered d-Band Center for Oxygen Reduction to Hydrogen Peroxide in Alkaline Solution

Chaoqi Zhang,[1] Ruihu Lu,[4] Chao Liu,[1]* Jingyi Lu,[1] Yingying Zou,[1] Ling Yuan,[1] Jing Wang,[1] Guozhong Wang,[3] Yan Zhao, [4]* Chengzhong Yu*[1, 2]

[1]C. Q. Zhang, Prof. C. Liu, J.Y. Lu, Y. Y. Zou, L. Yuan, Dr. J. Wang, Prof. C. Z. Yu

School of Chemistry and Molecular Engineering, East China Normal University, Shanghai 200241, P. R. China.

*E-mail: cliu@chem.ecnu.edu.cn; czyu@chem.ecnu.edu.cn.

[2]Prof. C. Z. Yu

Australian Institute for Bioengineering and Nanotechnology, The University of Queensland, Brisbane, Queensland 4072, Australia.

*E-mail: c.yu@uq.edu.au.

[3]Prof. G. Z. Wang

Key Laboratory of Materials Physics, Centre for Environmental and Energy Nanomaterials, Anhui Key Laboratory of Nanomaterials and Nanotechnology, Institute of Solid State Physics, Chinese Academy of Sciences, Hefei 230031, P. R. China.

[4]R. H. Lu, Prof. Y. Zhao

State Key Laboratory of Silicate Materials for Architectures, Wuhan University of Technology, Wuhan 430070, China.

*E-mail: Yan2000@whut.edu.cn.

Experimental section

Chemicals

Titanium isopropoxide (TPOT, 97%, Aldrich), aminoterephthalate (ATA, 99%, Aldrich), cobalt nitrate hexahydrate ($Co(NO_3)_2·6H_2O$, Sinopharm Chemical Regent Co., Ltd), zinc nitrate htxahydeate ($Zn(NO_3)_2·6H_2O$, Sinopharm Chemical Regent Co., Ltd), 2-methylimidazole (2-MeIM, Aldrich), Cetyltrimethylammonium Bromide (CTAB, Adamas-beta), N,N-dimethylformamide (DMF, 99%, Greagent), thioacetamide (TAA, Adamas, 99%), ethanol (AR, 99%, Adamas-beta) and methanol (AR, 99%, Adamas-beta) were used as received. Deionized water was used in all experiments.

Synthesis of NH_2-MIL-125

Typically, 0.3 mL of TPOT and 0.28 g of ATA were added into 20 mL of mixture of DMF and CH_3OH (9:1, v/v). The solution was transferred into a 50 mL Teflon-line autoclave after vigorous stirring for 5 min, then heated at 150 °C for 24 h. The yellow solid product was collected by washing with DMF and methanol for three times. Finally, the obtained NH_2-MIL-125 was dispersed in 15 mL of methanol to make a stock solution (~20 mg/mL) for further use.

Synthesis of NH_2-MIL-125@ZnCo-ZIF heterostructure

NH_2-MIL-125@ZnCo-ZIF was synthesized as follows: 0.3 mL of NH_2-MIL-125 methanol dispersion, 5 mL of 2-MeIM solution (6.4 mg/mL) and 3 mL of $Co(NO_3)_2·6H_2O/Zn(NO_3)_2·6H_2O$ (5.7:1, w/w) solution (7.4 mg/mL) were mixed under gentle stirring for 2 h. The product was collected by centrifugation, washed with methanol three times and dried overnight.

Synthesis of Ti-ZnCoS HSS

In a typical synthesis, 10 mg of the as-synthesized NH_2-MIL-125@ZnCo-ZIF was dispersed in 10 mL of ethanol. 10 mL of thioacetamide ethanol solution (12.5 mg/mL) was then added into the above suspension under stirring for 10 minutes. Afterwards, the mixture was transferred into a 50 mL Teflon-line autoclave and kept at 180 °C for 3 h. The products were collected by centrifugation, washed with ethanol for five times and dried overnight, resulting in the Ti-ZnCoS HSS.

Synthesis of CoS_x and ZnCoS

In a typical method, 50 mL of 2-MeIM solution (6.4 mg/mL) was mixed with 30 mL of $Co(NO_3)_2·6H_2O$ or $Co(NO_3)_2·6H_2O/Zn(NO_3)_2·6H_2O$ (5.7/1, w/w) solution (7.4 mg/mL) with stirring for 2 h to form ZIF-67 or ZnCo-ZIF. The product was collected by centrifugation, washed with methanol for three times and dried at 45 °C for 24 h. Subsequently, similar

sulfidation process with Ti-ZnCoS HSS was used to convert ZIF-67 and ZnCo-ZIF into corresponding metal sulfides, denoted as CoS_x and ZnCoS.

Synthesis of Ti-ZnCoS HP

In a typical method, 25 mg of synthesized ZnCo-ZIF and 5 mg of NH_2-MIL-125 were dispersed in 30 mL of ethanol solution. To this dispersion, 30 mL of thioacetamide ethanol solution (12.5 mg/mL) was added, then the mixture was transferred into a 100 mL Teflon-line autoclave and kept at 180 °C for 3 h. The product was collected by centrifugation, washed with ethanol for three times and dried overnight.

Synthesis of ZnS

ZIF-8 was first synthesized according to the previous reported method.[1] Typically, 10.08 g of 2-methlomedizole was dissolved in 170 mL of deionized water. 4.5 mL of 0.01 M CTAB was added into above solution with stirring for 5 min. Afterwards, 30 mL of $Zn(NO_3)_2{\cdot}6H_2O$ solution (23.7 mg/mL) was added into the mixture solution with stirring for 4 h at room temperature. The white product was collected by centrifugation, then washed with methanol for three times before drying at 60 °C for 5 h. To synthesize ZnS, 40 mg of ZIF-8 was dispersed in 40 mL of TAA ethanol solution (7 mg/mL), which was then refluxed at 95 °C for 1 h. The ZnS solid was collected by centrifugation, washed by ethanol for three times and finally dried overnight.

Synthesis of Ti-ZnCoS HSS-1

Ti-ZnCoS HSS-1 was prepared using the same process with Ti-ZnCoS HSS except for changing the $Co(NO_3)_2{\cdot}6H_2O/Zn(NO_3)_2{\cdot}6H_2O$ ratio into 19/1.

Characterization

Wide-angle X-ray diffraction (XRD) patterns were recorded on a Bruker D8 Advance Powder X-ray diffractometer (Bruker AXS, Germany) operating at 40 mA and 40 kV with Cu-Kα radiation source. X-ray photoelectron spectroscopy (XPS) studies were carried out on a Thermo ESCALAB 250 using an Al Kαradiation and C 1s (284.8 eV) as a reference to correct the binding energy. Transmission electron microscopy (TEM) and high-resolution transmission electron microscopy (HRTEM) images were collected on a JEM-2100F (JEOL, Japan) with an acceleration voltage of 200 kV. Scanning electron microscopy (SEM) images were acquired by a scanning electron microscope (HITACHI-S4800). N_2 sorption isotherms were measured using Micromeritics ASAP-2460 at liquid nitrogen temperature (-196 °C). Raman spectra of the samples were measured on a GX-PT-1500 instrument with an incident wavelength of 532 nm at a power of around 1 mW.

Electrochemical measurement

Rotating ring-disk electrode (RRDE) tests were conducted in a standard three-electrode system on a CHI-760C electrochemical analyzer (CH Instruments Inc.) in O_2-saturated 0.1 M KOH solution at ambient temperature with the platinum wire as counter electrode, the Ag/AgCl (KCl, 3.5 M) as reference electrode and the catalyst-modified glassy carbon as working electrode. The catalyst inks were prepared by dispersing 10 mg sample into 1 mL of isopropanol containing 30 μL of Nafion solution to form a homogeneous suspension. The prepared ink was deposited on the polished glassy carbon disk with a mass loading of 0.61 mg cm^{-2} and dried under an infrared lamp.

The collection efficiency (N) was experimentally determined by $[Fe(CN)_6]^{3-/4-}$ redox reaction with N_2-saturated 0.1 M KNO_3+10 mM $K_3[Fe(CN)_6]$ solution as electrolyte. The chronoamperometry was performed at 0.5 V vs. RHE while the ring potential was fixed at 1.5 V vs. RHE for 200 s. The collection efficiency could be calculated as follows: $N = i_r / i_d$.

The RRDE was rotated at 1600 rpm throughout the whole tests. Liner sweep voltammetry (LSV) curves were recorded at a scan rate of 5 mV s^{-1}. The ring electrode was set to a constant voltage of 1.2 V vs. RHE.[2] The potential reaching the ORR current density of 1 mA cm^{-2} in rotating disk electrode (RDE) polarization curves was recorded to be the onset potential to evaluate the reactivity of catalysts.[3] The selectivity of H_2O_2 was calculated using the following equation:

$$H_2O_2(\%) = 200 \times (\frac{i_r}{N})/(\frac{i_r}{N} + i_d)$$

where i_r is the ring current, i_d is the disk current and N is the current collection efficiency of the Pt ring electrode (N=0.258). The number of electron transferred (n) in the oxygen reduction reaction (ORR) was obtained as following:

$$n = \frac{4i_d}{i_d + i_r/N}$$

The Tafel slope (b) was obtained by fitting the linear part of the Tafel plots according to the Tafel equation (η =a+b log (j)) to evaluate the kinetic performance of as-prepared catalysts for ORR.

The electrochemical active areas (ECSA) were calculated by Parsons-Zobel plot method. Within the range of the potential where no electrochemical reaction occurs, the limiting current density (J) and scanning rate (v) present the following functional relationship:

$$J = vC_{dl},$$

where C_{dl} is the double-layer capacitance. ECSA was further given through the following equation:

$$\mathrm{ECSA} = \frac{C_{dl}}{A \times C_s},$$

where A is the amount of the material coating on the surface of electrode ($mg \cdot cm^{-2}$), Cs is an empirical constant representing the capacitance per unit area ($40mF \cdot cm^{-2}$). Electrochemical impedance spectroscopy (EIS) was measured in 0.1 M KOH solution in the frequency range of 1000 kHz to 0.01 Hz with an amplitude of 10 mV.

The cumulative H_2O_2 yield measurement was conducted in H-type electrolytic cell separated by Nafion 117 membrane with Ag/AgCl (KCl, 3.5 M) as the reference electrode, graphite rod as the counter electrode and the Ti-ZnCoS HSS modified carbon fiber paper (1 cm×1.3 cm) as the working electrode with a mass loading of 0.2 mg cm^{-2}. 70 mL of electrolyte was placed in a electrolytic cell with volume of 100 mL. The desired constant voltage was carried out for 90 min and the concentration of generated H_2O_2 was detected by iodometry. Typically, 100 μL of reaction solution was collected from the electrochemical system and subsequently added to the mixture of potassium hydrogen phthalate ($C_8H_5KO_4$) and potassium iodide (KI) aqueous solution with reaction for 30 min. The H_2O_2 molecules will react with I^- to generate I^{3-} ($H_2O_2 + I^- \rightarrow I^{3-} + H_2O$). The amount of I^{3-} was measured by a Synergy-H1 microplate reader at its characteristic absorbance peak of 350 nm for H_2O_2 quantification.

The stability of catalyst was tested with the chronoamperometric technique at a constant voltage of 0.6 V vs RHE. All the potentials were calibrated with a reversible hydrogen electrode (RHE) ($E_{RHE}= E_{Ag/AgCl}+0.0591\times pH+0.197$).

To prepare the solid H_2O_2, the saturated $NaCO_3$ solution with 0.1% wt. stabilizer ($NaSiO_3$: EDTA = 3:1) was added to the electrolyte dropwise within 15min. After 1h reaction, sodium chloride was added as salting-out agent and the mixture solution was placed in the ice bath to cool and crystallize. The white powder was collected by vacuum filtration, washed by isopropyl and vacuum dried overnight.[4]

Computational details

The Shirley background is subtracted from the measured spectra. The position of the center of the valence band (d-band center) is given by following equation:

$$E_d = \int N(\varepsilon)\varepsilon \, d\varepsilon \,/\, \int N(\varepsilon) \, d\varepsilon$$

Where N(ε) is the DOS or, in our case, the XPS-intensity after background subtraction.

Density functional theory (DFT) calculations were conducted using the Vienna Ab initio simulation package (VASP).[5-7] The popular Perdew-Burke-Ernzerhof (PBE)[8] functional is employed for the exchange-correlation energies, and it is a generalized gradient approximation (GGA). The interaction between core electrons and valence electrons is described by the frozen-core projector-augmented wave (PAW)[9, 10] method with a cut off energy of 500 eV. Furthermore, the long-range van der Waals (vdW) interactions are calculated with Grimme's DFT-D3 method.[11] The construction of the models adopts the amorphous structure, which is confirmed by the XRD results. The amorphous nanoparticle of CoS_x consisting of 64 atoms with a radius of 11 Å. Furthermore, Zn-, Ti-doping were adopted to replace the Co atom in the CoS_x nanocluster with the Zn and/or Ti atoms. A 10 Å vacuum layer is used to eliminate the interactions with the periodic images. And a Γ-centred Monkhorst-Pack *k*-point mesh grid of 1 × 1 × 1 is employed for all structural optimizations.[12] Moreover, the criteria of energy and force convergence are set to 1.0×10^{-5} eV per atom and 0.01 eV $Å^{-1}$, respectively, for geometry optimization.

The widely accepted theoretical method to stimulate the ORR reaction is a two-electron transfer reaction pathway in a basic medium, as shown in Eqs. 1 and 2:

$$* + O_2 + H_2O + e^- \rightarrow *OOH + OH^- \quad (1)$$

$$*OOH + e^- \rightarrow * + OOH^- \quad (2)$$

where *, *OOH, *O, and *OH denote adsorbed sites, adsorbed intermediates OOH, *O, and *OH, respectively.

For each element step, the Gibbs free energy ΔG can be calculated using Eq. 3.

$$\Delta G = \Delta E + \Delta E_{ZPE} - T\Delta S \quad (3)$$

where ΔE is the total energy of reactions obtained from DFT calculations, ΔE_{ZPE} and ΔS represent the zero-point energy and entropic changes, respectively. T is the temperature (298.15 K). According to the computational hydrogen electrode (CHE) model proposed by Nørskov et al.,[13] the energy involving H_2O/OH^- electron in Eq. 1 and 2 is equal to the half of an H_2 molecule. Because of the difficulties in the DFT calculations of open-shell triplet O_2, the free energy of O_2(g) is obtained by $G_{O_2(g)} = 2G_{H_2O} - 2G_{H_2} + 4.92$ eV.[14]

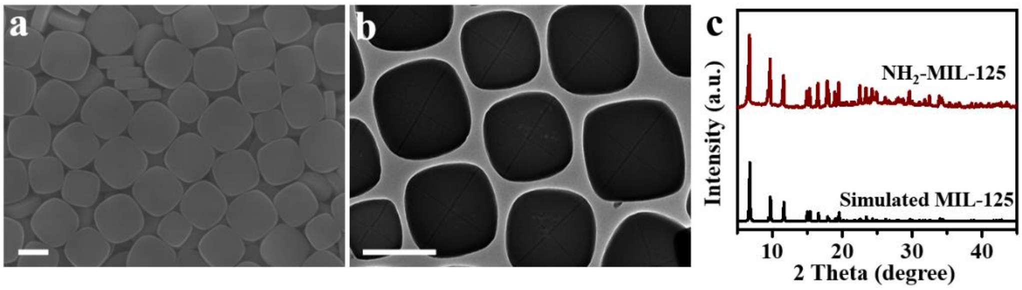

Figure S1. (a) SEM image, (b) TEM image and (c) XRD pattern of NH_2-MIL-125. Scale bar is 500 nm.

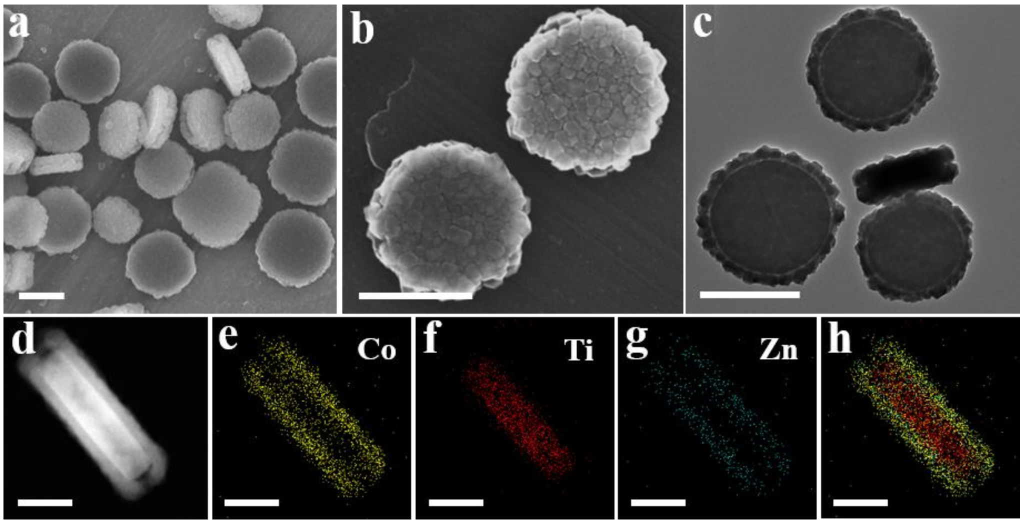

Figure S2. (a, b) SEM images, (c) TEM image and (d-h) HADDF-STEM and element mapping images of NH_2-MIL-125@ZnCo-ZIF. Scale bars are (a, b and c) 500 nm, (d-h) 200 nm.

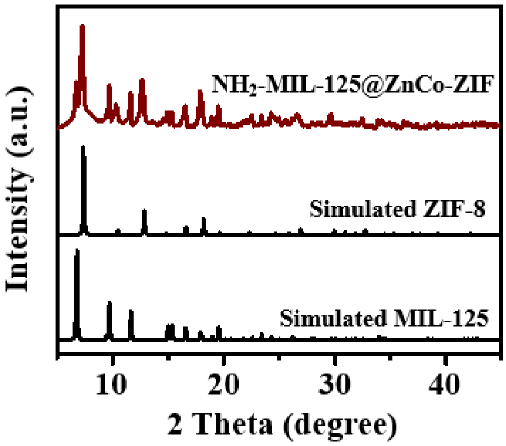

Figure S3. XRD pattern of NH_2-MIL-125@ZnCo-ZIF.

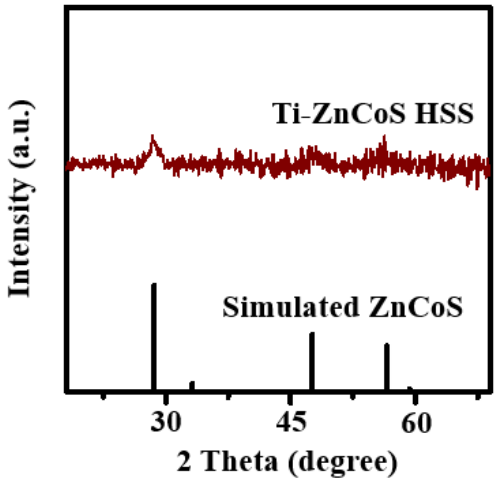

Figure S4. XRD pattern of Ti-ZnCoS HSS.

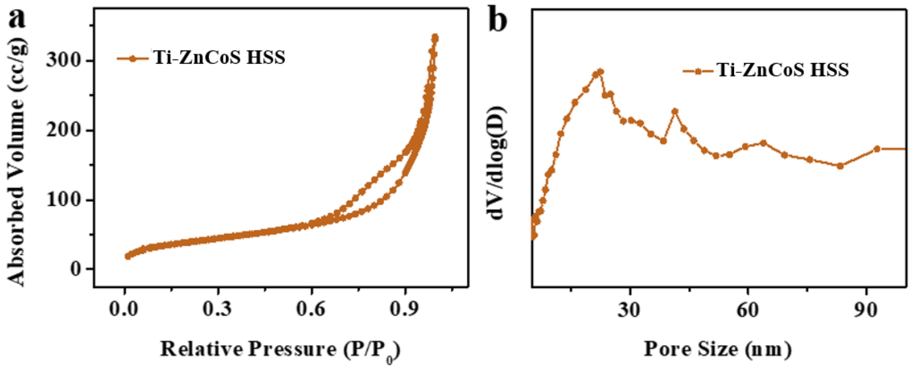

Figure S5. (a) N_2 sorption isotherms and (b) pore size distribution curve of Ti-ZnCoS HSS

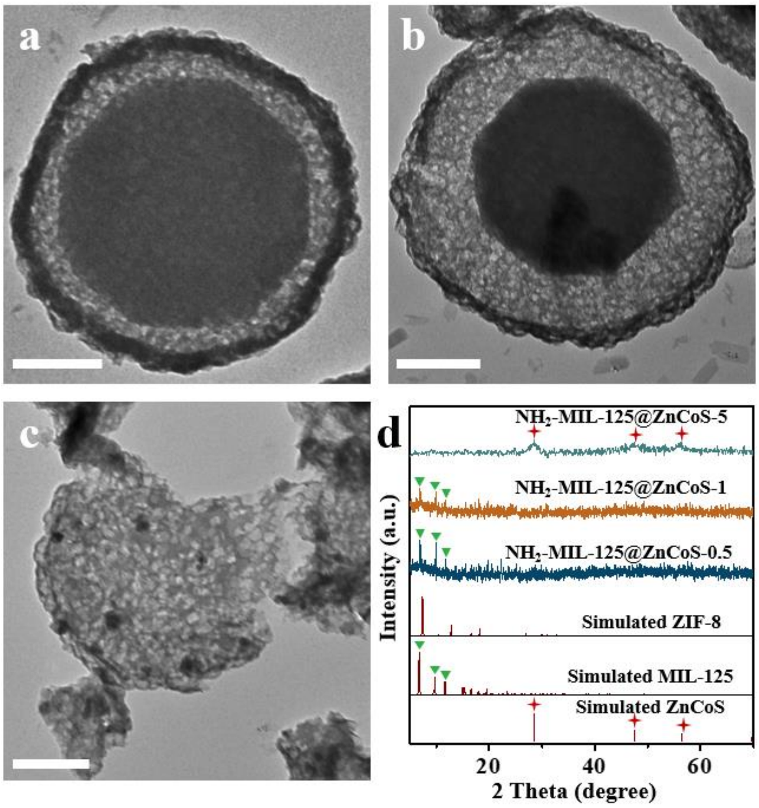

Figure S6. (a-c) TEM images and (d) XRD patterns of NH_2-MIL-125@ZnCoS-0.5, -1 and -5. Scale bar is 200 nm.

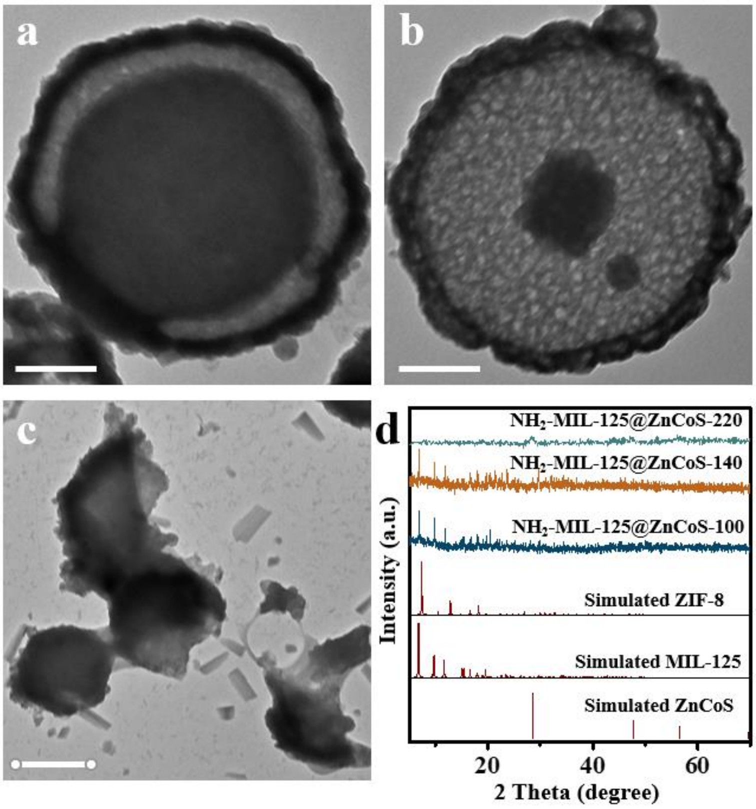

Figure S7. (a-c) TEM images and (d) XRD patterns of NH_2-MIL-125@ZnCoS-100, -140 and -220. Scale bar is 200 nm.

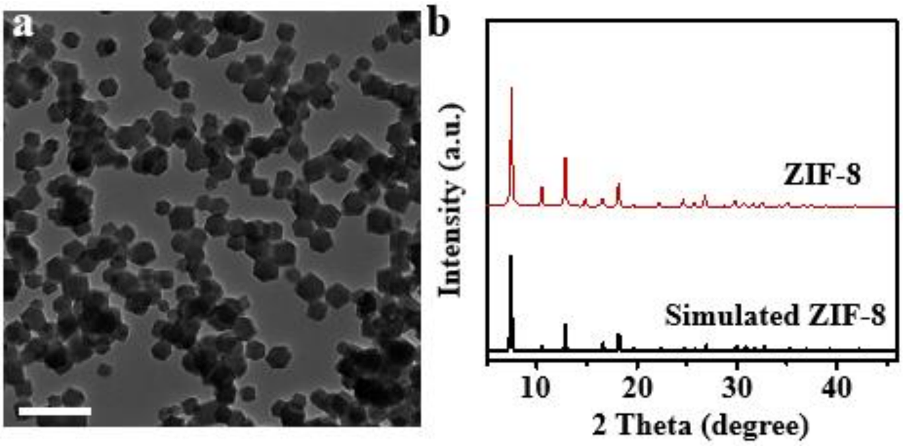

Figure S8. (a) TEM image and (b) XRD pattern of ZIF-8. Scale bar is 200 nm.

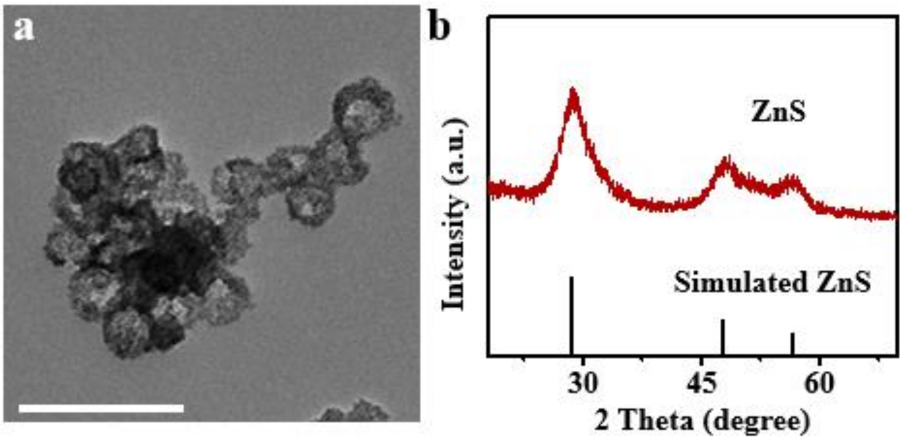

Figure S9. (a) TEM image and (b) XRD pattern of ZnS. Scale bar is 200 nm.

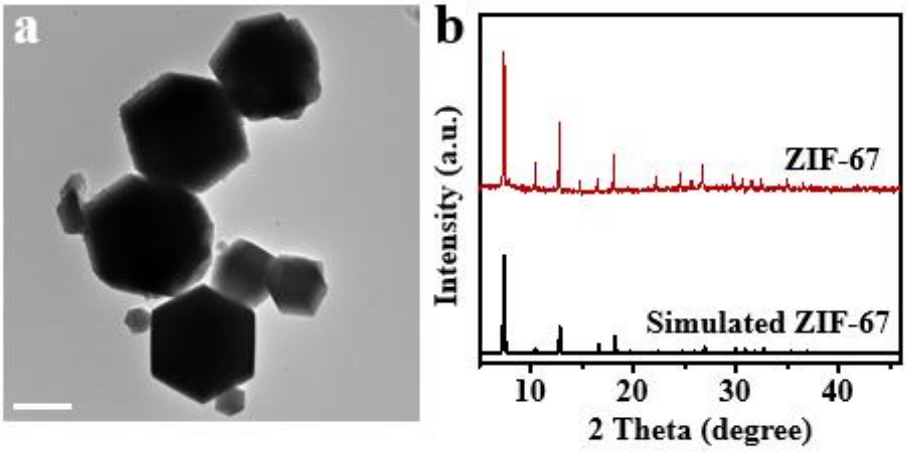

Figure S10. (a) TEM image and (b) XRD pattern of ZIF-67. Scale bar is 1 μm.

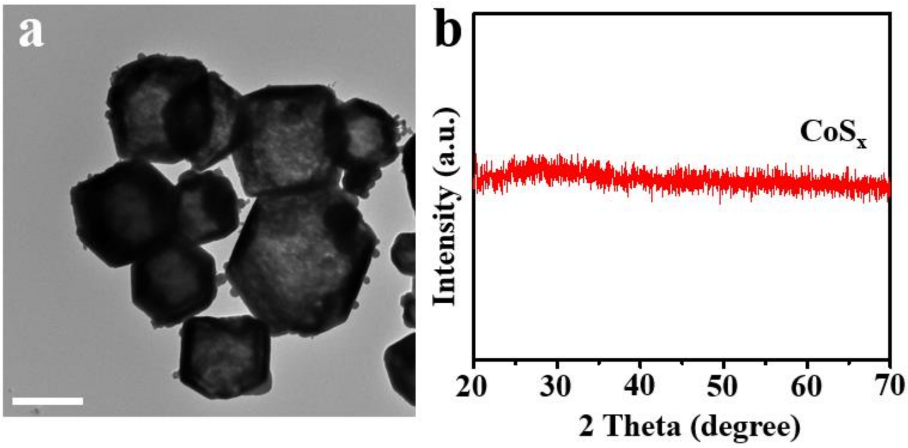

Figure S11. (a) TEM image and (b) XRD pattern of CoS_x. Scale bar is 1 μm.

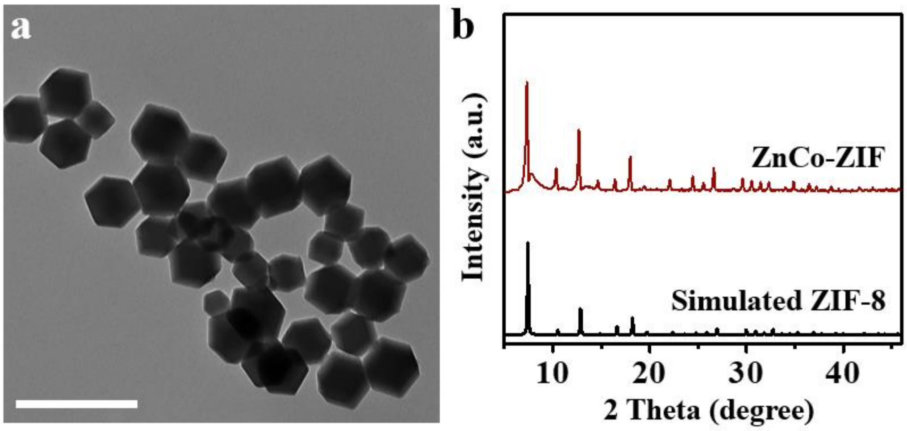

Figure S12. (a) TEM image and (b) XRD pattern of ZnCo-ZIF. Scale bar is 500 nm.

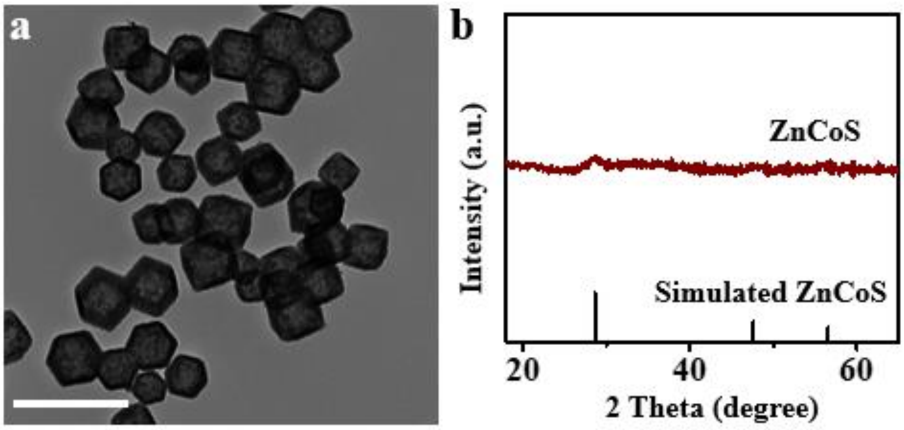

Figure S13. (a) TEM image and (b) XRD pattern of ZnCoS. Scale bar is 500 nm.

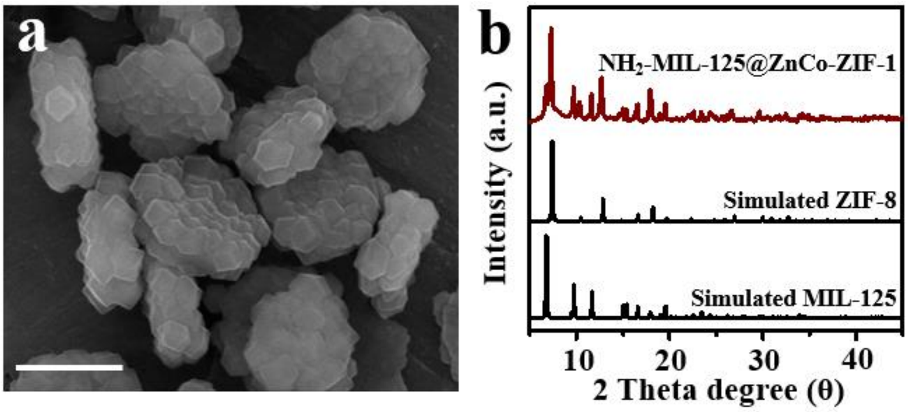

Figure S14. (a) SEM image and (b) XRD pattern of NH_2-MIL-125@ZnCo-ZIF-1. Scale bar is 500 nm.

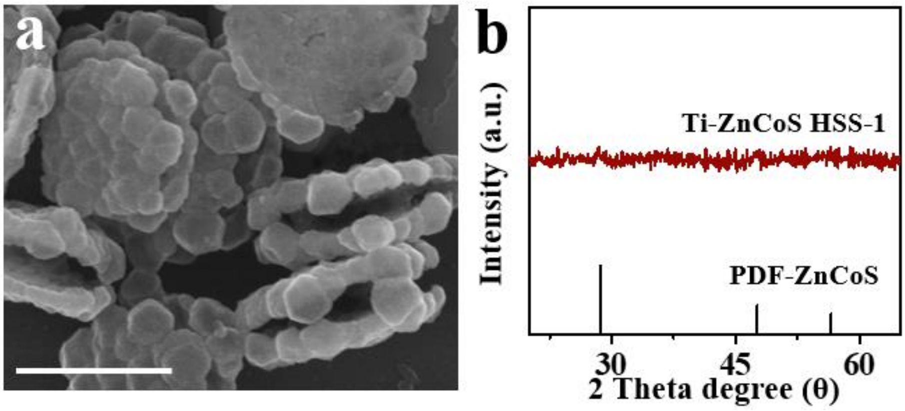

Figure S15. (a) SEM image and (b) XRD pattern of Ti-ZnCoS HSS-1. Scale bar: 500 nm.

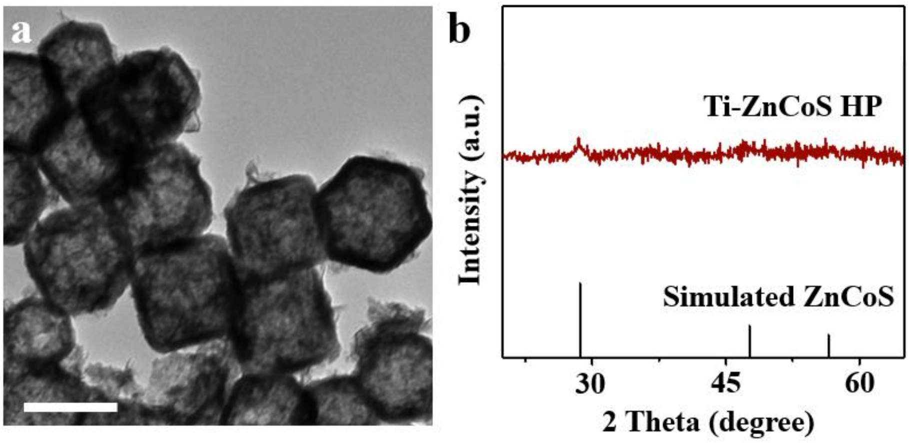

Figure S16. (a) TEM image and (b) XRD pattern of Ti-ZnCoS HP. Scale bar is 200 nm.

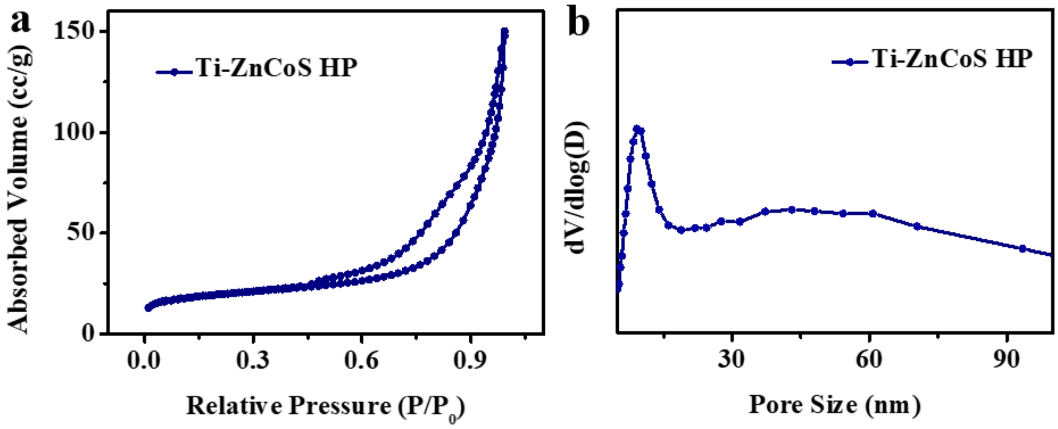

Figure S17. (a) N_2 sorption isotherms and (b) pore size distribution curve of Ti-ZnCoS HP.

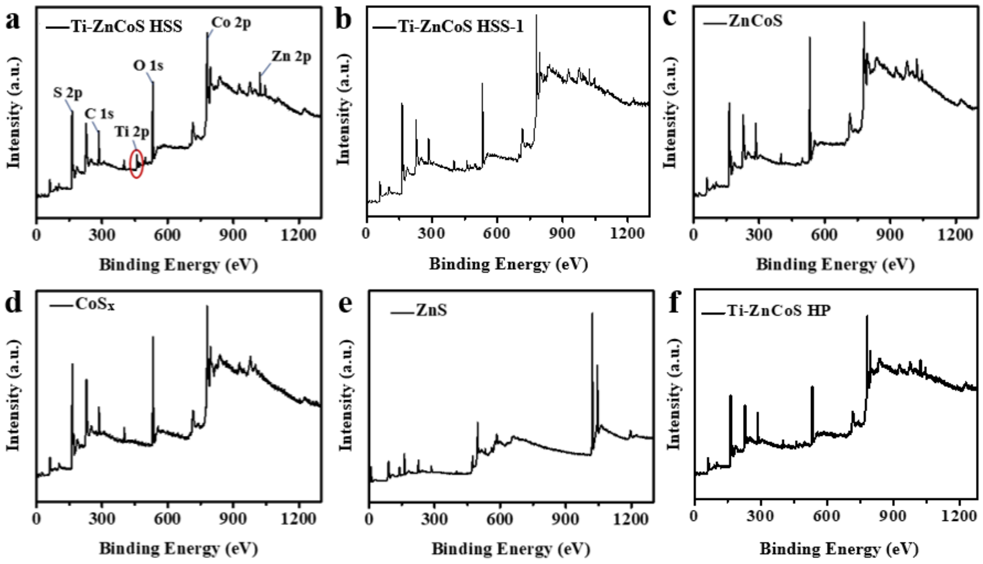

Figure S18. XPS spectra of Ti-ZnCoS HSS (a), Ti-ZnCoS HSS-1 (b), ZnCoS (c), CoS_x (d), ZnS (e) and Ti-ZnCoS HP (f).

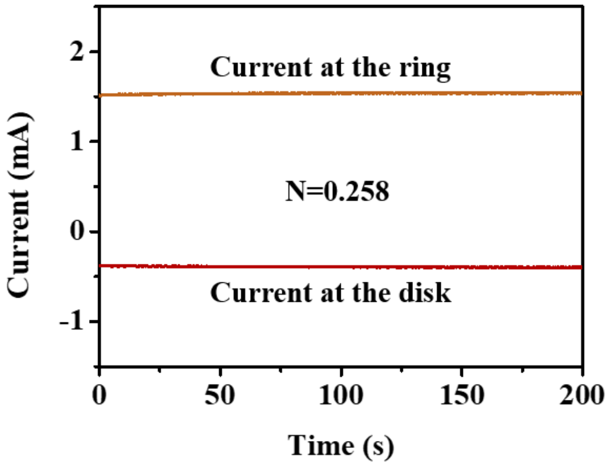

Figure S19. The collection efficiency test.

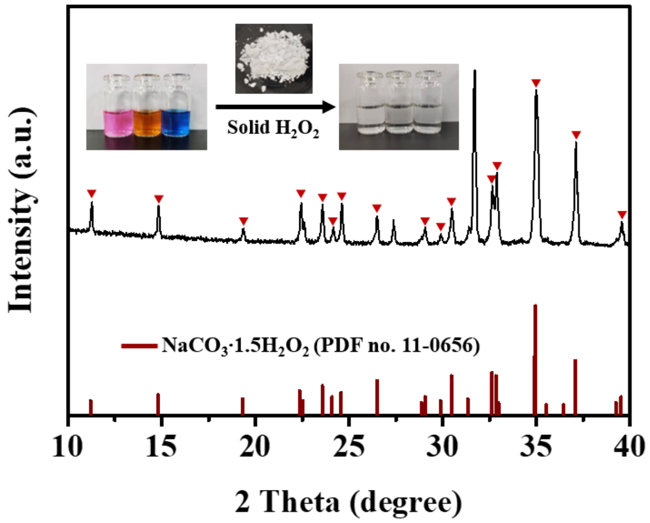

Figure S20. The XRD pattern of as-extracted solid H_2O_2 from electrolyte. The inset shows the degradation of organic dyestuff with solid H_2O_2.

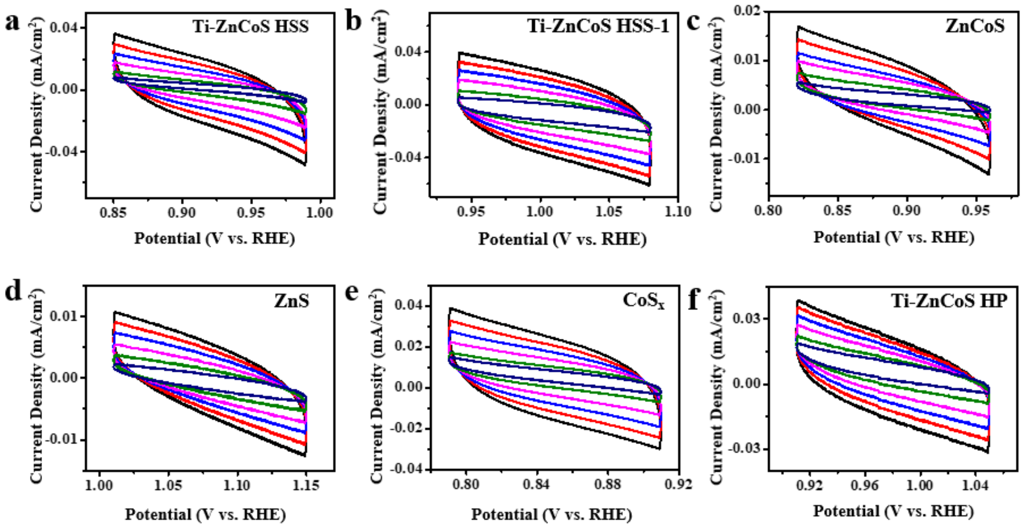

Figure S21. CV curves of Ti-ZnCoS HSS, Ti-ZnCoS HSS-1, Ti-ZnCoS HP, ZnCoS, ZnS and CoS_x at different scan rates.

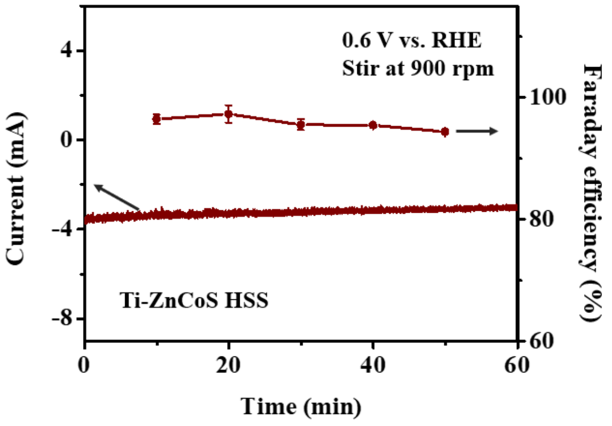

Figure S22. Bulk ORR electrolysis of Ti-ZnCoS HSS/CFP electrode and cumulative H_2O_2 Faraday efficiency.

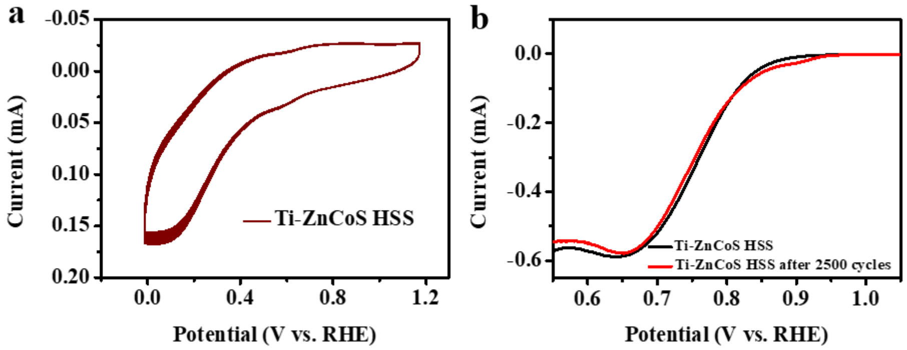

Figure S23. (a) 2500 CV cycles and (b) LSV curves of Ti-ZnCoS HSS before and after 2500 cycles.

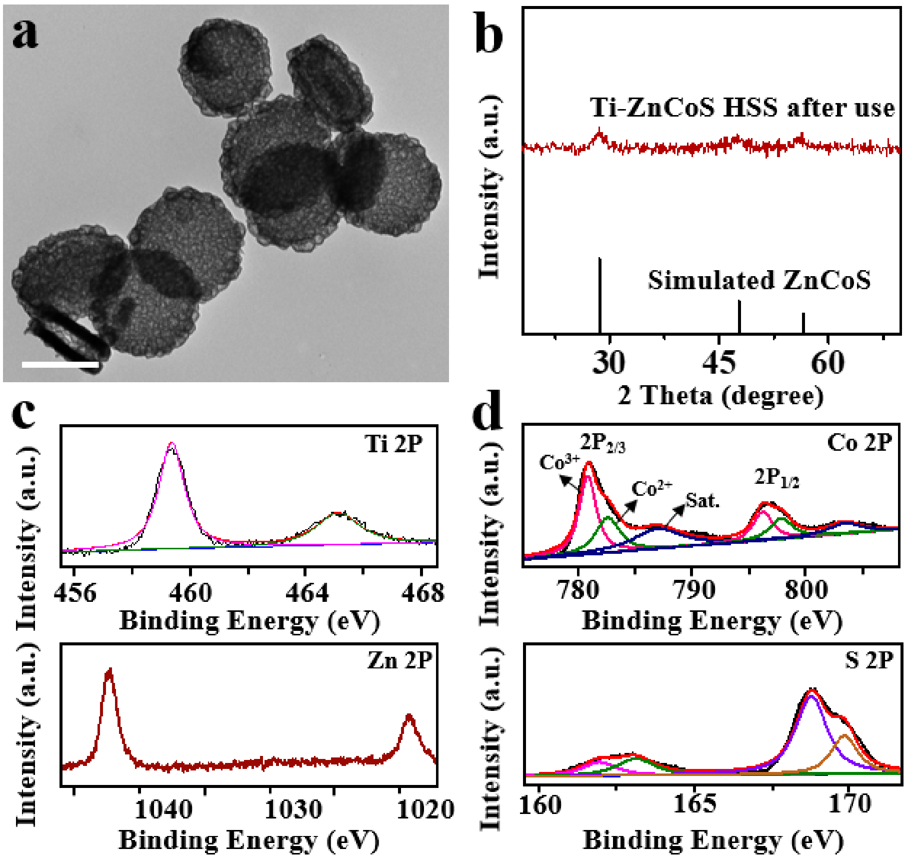

Figure S24. (a) TEM image, (b) XRD pattern and (c, d) High resolution XPS spectra of Ti 2p, Co 2p, Zn 2p and S 2p of Ti-ZnCoS HSS after 12 hours test at 0.6 V vs.RHE. Scale bar is 500 nm.

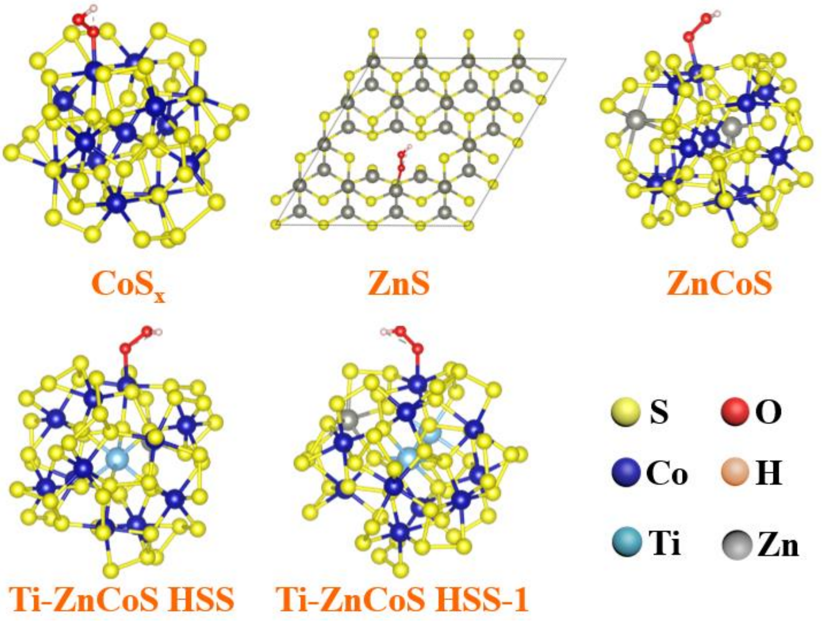

Figure S25. Structural models of CoS_x, ZnS, ZnCoS, Ti-ZnCoS HSS and Ti-ZnCoS HSS-1.

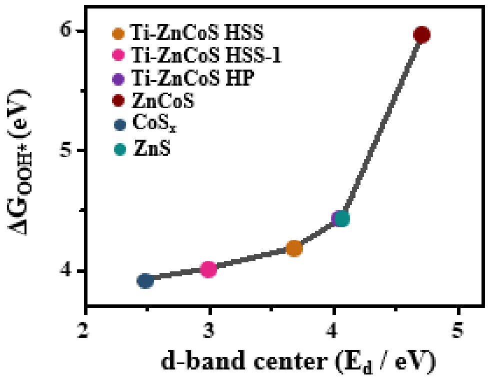

Figure S26. The relationship between the calculated ΔG_{OOH*} and d-band centers of CoS_x, ZnS, ZnCoS, Ti-ZnCoS HSS and Ti-ZnCoS HSS-1.

Table S1. ICP results of Ti-ZnCoS HP and Ti-ZnCoS HSS.

Catalysts	Content (mg/g)		
	Ti	Zn	Co
Ti-ZnCoS HSS	47.2	47.7	179.6
Ti-ZnCoS HP	47.6	54.5	190.2

Table S2. The reported 2e-ORR electrocatalysts in alkaline condition.

Sample	Onset potential	Selectivity	Productivity	Electrolyte	Ref.

	**(V vs. RHE)**	**(%)**			
Ti-ZnCoS HSS	0.79	98	675 mmol h^{-1} g_{cat}^{-1}	0.1M KOH	This work
Co-POC-O	0.78	84	831 mg L^{-1} h^{-1}	0.1M KOH	3
O-CNT	0.70	90	3950 mg L^{-1} h^{-1}	0.1 M KOH	14
Fe_3O_4/graphehe	0.76	63	-	1 M KOH	15
Printex L6 A	0.73	92	-	1 M NaOH	16
Printex L6	0.54	88	-	1 M NaOH	17
N-FLG	0.73	70	9.66 mol h^{-1} g_{cat}^{-1}	0.5 M KOH	18
g-N-CNHs	0.53	90	-	0.1 M NaOH	19
CMK3-20	0.79	90	2.476 mol h^{-1} g_{cat}^{-1}	0.1 M KOH	20
Au–Pt–Ni	0.7	95	2.22 mg L^{-1} h^{-1}	0.1 M KOH	21
GC400	0.73	93	-	0.1 M KOH	22
α-Fe_2O_3	0.62	95	454 mmol h^{-1} g_{cat}^{-1}	0.1 M KOH	23
S-NiP_4Mo_6	0.76	83.9	4.84 mol h^{-1} g_{cat}^{-1}	0.1 M KOH	24
NF-Cs	0.74	89.6	-	0.1 M KOH	25
Mo SAC	0.69	95	-	0.1 M KOH	26
a-TiO_{2-x}/TiC	0.75	94	7.19 mol g_{cat}^{-1} h^{-1}	0.1 M KOH	27
Bi_2Te_3NPs	0.61	100	6.86 mmol L^{-1} h^{-1}	0.1 M KOH	28
Co1-NG(O)	0.8	82	418 mmol g^{-1} h^{-1}	0.1 M KOH	29
Co-N-C	0.82	60	~4.33 mol g_{cat}^{-1} h^{-1}	0.1 M KOH	30
GO/H_2O_2/$NH_3{\cdot}H_2O$	0.76	82	0.225 mol g_{cat}^{-1} h^{-1}	0.1 M KOH	31
HPCS-S	-	70	0.184 mol g_{cat}^{-1} h^{-1}	0.1 M KOH	32
rGO/PEI	0.76	90.7	0.106 mol g_{cat}^{-1} h^{-1}	0.1 M KOH	33
G-COF-950	0.74	75	1.287 mol g_{cat}^{-1} h^{-1}	0.1 M KOH	34
Ni-N_2O_2/C	-	96	5.9 mol g_{cat}^{-1} h^{-1}	0.1 M KOH	35
$OCNS_{900}$	0.83	90	770 mmol g^{-1} h^{-1}	0.1 M KOH	36
Ni MOF NSs	0.75	94	80 mmol g_{cat}^{-1} h^{-1}	0.1 M KOH	37

Table S3. The slopes of samples in low frequency of Nyquist plots

Samples	Slopes
CoS_x	1.94
ZnS	3.08
ZnCoS	0.74
Ti-ZnCoS HSS	3.59
Ti-ZnCoS HSS-1	2.22
Ti-ZnCoS HP	1.61

Reference

1. C. Liu, Q. Sun, L. Lin, J. Wang, C. Zhang, C. Xia, T. Bao, J. Wan, R. Huang, J. Zou, C. Yu. Ternary MOF-on-MOF heterostructures with controllable architectural and compositional complexity via multiple selective assembly. *Nat. Commun.* **2020**, *11*, 4971.
2. H. W. Kim, M. B. Ross, N. Kornienko, L. Zhang, J. Guo, P. Yang, B. D. McCloskey. Efficient hydrogen peroxide generation using reduced graphene oxide-based oxygen reduction electrocatalysts. *Nat. Catal.* **2018**, *1*, 282-290.
3. B.-Q. Li, C.-X. Zhao, J.-N. Liu, Q. Zhang. Electrosynthesis of hydrogen peroxide synergistically catalyzed by atomic Co-N_x-C sites and oxygen functional groups in noble-metal-free electrocatalysts. *Adv. Mater.* **2019**, *31*, 1808173.
4. McKillop, A. & Sanderson, W. R. Sodium perborate and sodium percarbonate: further applications in organic synthesis. *J. Chem. Soc. Perkin Trans. I.* **2020**, *1*, 471-476.
5. G. Kresse, J. Furthmüller. Efficiency of ab-initio total energy calculations for metals and semiconductors using a plane-wave basis set. *Computational Materials Science.* **1996**, *6*, 15-50.
6. G. Kresse, J. Furthmüller. Efficient iterative schemes for ab initio total-energy calculations using a plane-wave basis set. *Phys. Rev. B.* **1996**, *54*, 11169-11186.
7. G. Kresse, J. Hafner. Ab initio molecular-dynamics simulation of the liquid-metal-amorphous-semiconductor transition in germanium. *Phys. Rev. B.* **1994**, *49*, 14251-14269.
8. J. P. Perdew, K. Burke, M. Ernzerhof. Generalized Gradient Approximation Made Simple. *Phys. Rev. Lett.* **1996**, *77*, 3865-3868.
9. G. Kresse, D. Joubert. From ultrasoft pseudopotentials to the projector augmented-wave method. *Phys. Rev. B.* **1999**, *59*, 1758-1775 (1999).
10. P. E. Blöchl. Projector augmented-wave method. *Phys. Rev. B.* **1994**, *50*, 17953-17979.
11. S. Grimme, J. Antony, S. Ehrlich, H. Krieg. A consistent and accurate ab initio parametrization of density functional dispersion correction (DFT-D) for the 94 elements H-Pu. *J. Chem. Phys.* **2010**, *132*, 154104.
12. H. J. Monkhorst, J. D. Pack. Special points for Brillouin-zone integrations. *Phys. Rev. B.* **1976**, *13*, 5188-5192.
13. J. K. Nørskov, J. Rossmeisl, A. Logadottir, L. Lindqvist, J. R. Kitchin, T. Bligaard, H. Jónsson. Origin of the Overpotential for Oxygen Reduction at a Fuel-Cell Cathode. *J. Phys. Chem. B.* **2004**, *108*, 17886-17892.
14. Z. Lu, G. Chen, S. Siahrostami, Z. Chen, K. Liu, J. Xie, L. Liao, T. Wu, D. Lin, Y. Liu, T.

F. Jaramillo, J. K. Nørskov, Y. Cui. High-efficiency oxygen reduction to hydrogen peroxide catalysed by oxidized carbon materials. *Nat. Catals.* **2018**, *1*, 156-162.
15. W. R.P. Barros, Q. Wei, G. Zhang, S. Sun, M. R.V. Lanza, A. C. Tavares. Oxygen reduction to hydrogen peroxide on Fe_3O_4 nanoparticles supported on Printex carbon and Graphene. *Electrochim. Acta.* **2015**, *162*, 263-270.
16. M. H. M. T. Assumpção, R. F. B. D. Souza, D. C. Rascio, J. C. M. Silva, M. L. Calegaro, I. Gaubeur, T. R. L. C. Paixão, P. Hammer, M. R. V. Lanza, M. C. Santos. A comparative study of the electrogeneration of hydrogen peroxide using Vulcan and Printex carbon supports. *Carbon.* **2011**, *49*, 2842-2851.
17. A. Moraes, M. H. M. T. Assumpção, F. C. Simões, V. S. Antonin, M. R. V. Lanza, P. Hammer, M. C. Santos. Surface and catalytical effects on treated carbon materials for hydrogen peroxide electrogeneration. *Electrocatal.* **2016**, *7*, 60-69.
18. L. Li, C. Tang, Y. Zheng, B. Xia, X. Zhou, H. Xu, S.-Z. Qiao. Tailoring selectivity of electrochemical hydrogen peroxide generation by tunable pyrrolic-nitrogen-carbon. *Adv. Energy. Mater.* **2020**, *10*, 2000789.
19. D. Iglesias, A. Giuliani, M. Melchionna, S. Marchesan, A. Criado, L. Nasi, M. Bevilacqua, C. Tacagnacco, F. Vizza, M. Prato, P. Fornasiero. N-Doped graphitized carbon nanohorns as a forefront electrocatalyst in highly selective O_2 Reduction to H_2O_2. *Chem.* **2018**, *4*, 106-123.
20. Z. Chen, S. Chen, S. Siahrostami, P. Chakthranont, C. Hahn, D. Nordlund, S. Dimosthenis, J. K. Nørskov, Z. Bao, T. F. Jaramillo. Development of a reactor with carbon catalysts for modular-scale, low-cost electrochemical generation of H_2O_2. *React. Chem. Eng.* **2017**, *2*, 239-245.
21. Z. Zheng, Y. H. Ng, D.-W. Wang, R. Amai. Epitaxial growth of Au-Pt-Ni nanorods for direct high selectivity H_2O_2 production. *Adv. Mater.* **2016**, *28*, 9949-9955.
22. Y.-H. Lee, F. Li, K.-H. Chang, C.-C. Hu, T. Ohsaka. Novel synthesis of N-doped porous carbons from collagen for electrocatalytic production of H_2O_2. *Appl. Catal. B.* **2012**, *126*, 208-214.
23. R. Gao, L. Pan, Z. Li, C. Shi, Y. Yao, X. Zhang, J.-J. Zou. Engineering facets and oxygen vacancies over hematite single crystal for intensified electrocatalytic H_2O_2 production. *Adv. Funct. Mater.* **2020**, *30*, 1910539.
24. Y. Zheng, X. Xu, J. Chen, Q. Wang. Surface O^{2-} regulation on POM electrocatalyst to achieve accurate 2e/4e-ORR control for H_2O_2 production and Zn-air battery assemble. *Appl. Catal. B.* **2021**, *285*, 119788.
25. N. Jia, T. Yang, S. Shi, X. Chen, Z. An, Y. Chen, S. Yin, P. Chen. N,F-codoped carbon nanocages: an efficient electrocatalyst for hydrogen peroxide electroproduction in alkaline and acidic solutions. *ACS Sustain. Chem. Eng.* **2020**, *8*, 2883-2891.
26. C. Tang, Y. Jiao, B. Shi, J.-N. Liu, Z. Xie, X. Chen, Q. Zhang, S.-Z. Qiao. Coordination tunes selectivity: two-electron oxygen reduction on high-loading molybdenum single-atom catalysts. *Angew. Chem. Int. Ed.* **2020**, *59*, 9171-9176.
27. Z. Xu, J. Liang, Y. Wang, K. Dong, X. Shi, Q. Liu, Y. Luo, T. Li, Y. Jia, A. M. Asoro, Z. Feng, Y. Wang, D. Ma, X. Sun. Enhanced Electrochemical H_2O_2 Production via Two-Electron Oxygen Reduction Enabled by Surface-Derived Amorphous Oxygen-Deficient TiO_{2-x}. *ACS. Appl. Mater. Interfaces.* **2021**, *13*, 33182-33187.
28. N. Zhang, F. Zheng, B. Huang, Y. Ji, Q. Shao, Y. Li, X. Xiao, X. Huang. Exploring Bi_2Te_3 Nanoplates as Versatile Catalysts for Electrochemical Reduction of Small Molecules. *Adv. Mater.* **2020**, *32*, 1906477.
29. E. Jung, H. Shin, B.-H. Lee, V. Efremov, S. Lee, H. S. Lee, J. Kim, W. H. Antink, S. Park, K.-S. Lee, S.-P. Cho, J. S. Yoo, Y.-E. Sung, T. Hyeon. Atomic-level tuning of Co-N-C catalyst for high-performance electrochemical H_2O_2 production. *Nat. Mater.* **2020**, *19*, 436-442.

30. Y. Sun, L. Silvioli, N. R. Sahraie, W. Ju, J. Li, A. Zitolo, S. Li, A. Bagger, L. Arnarson, X. Wang, T. Moeller, D. Bernsmeier, J. Rossmeisl, F. Jaouen, P. Strasser. Activity-Selectivity Trends in the Electrochemical Production of Hydrogen Peroxide over Single-Site Metal-Nitrogen-Carbon Catalysts. *J. Am. Chem. Soc.* **2019**, *141*, 12372-12381.
31. L. Han, Y. Sun, S. Li, C. Cheng, C. E. Halbig, P. Ferchit, J. L. Hübner, P. Strasser, S. Eigler. In-Plane Carbon Lattice-Defect Regulating Electrochemical Oxygen Reduction to Hydrogen Peroxide Production over Nitrogen-Doped Graphene. *ACS Catal.* **2019**, *9*, 1283−1288.
32. G. Chen, J. Liu, Q. Li, P. Guan, X. Yu, L. Xing, J. Zhang, R. Che. A direct H_2O_2 production based on hollow porous carbon sphere-sulfur nanocrystal composites by confinement effect as oxygen reduction electrocatalysts. *Nano Research*. **2019**, *12*, 2614-2622.
33. X. Xiao, T. Wang, J. Bai, F. Li, T. Ma, Y. Chen. Enhancing the Selectivity of H2O2 Electrogeneration by Steric Hindrance Effect. *ACS Appl. Mater. Interfaces.* **2018**, *10*, 42534-42541.
34. J. Zhang, G. Zhang, S. Jin, Y. Zhou, Q. Ji, H. Lan, H. Liu, J. Qu. Graphitic N in nitrogen-Doped carbon promotes hydrogen peroxide synthesis from electrocatalytic oxygen reduction. *Carbon.* **2020**, *163*, 154-161.
35. Y. Wang, R. Shi, L. Shang, G. I. N. Waterhouse, J. Zhao, Q. Zhang, L. Gu, T. Zhang. High-Efficiency Oxygen Reduction to Hydrogen Peroxide Catalyzed by Nickel Single-Atom Catalysts with Tetradentate N_2O_2 Coordination in a Three-Phase Flow Cell. *Angew. Chem. Int. Ed.* **2020**, *59*, 13057-13062.
36. S. Chen, T. Luo, K. Chen, Y. Lin, J. Fu, K. Liu, C. Cai, Q. Wang, H. Li, X. Li, J. Hu, H. Li, M. Zhu, M. Liu. Chemical Identification of Catalytically Active Sites on Oxygen-doped Carbon Nanosheet to Decipher the High Activity for Electro-synthesis Hydrogen Peroxide. *Angew. Chem. Int. Ed.* **2021**, *60*, 16607-16614.
37. M. Wang, X. Dong, Z. Meng, Z. Hu, Y.-G. Lin, C.-K. Peng, H. Wang, C.-W. Pao, S. Ding, Y. Li, Q. Shao, X. Huang. An Efficient Interfacial Synthesis of Two-Dimensional Metal-Organic Framework Nanosheets for Electrochemical Hydrogen Peroxide Production. *Angew. Chem. Int. Ed.* **2021**, *60*, 11190-11195.
